# Oncogenic deubiquitination controls tyrosine kinase signaling and therapy response in acute lymphoblastic leukemia

**DOI:** 10.1126/sciadv.abq8437

**Published:** 2022-12-09

**Authors:** Qi Jin, Blanca Gutierrez Diaz, Tim Pieters, Yalu Zhou, Sonali Narang, Igor Fijalkwoski, Cristina Borin, Jolien Van Laere, Monique Payton, Byoung-Kyu Cho, Cuijuan Han, Limin Sun, Valentina Serafin, George Yacu, Wouter Von Loocke, Giuseppe Basso, Giulia Veltri, Ingrid Dreveny, Issam Ben-Sahra, Young Ah Goo, Stephanie L. Safgren, Yi-Chien Tsai, Beat Bornhauser, Praveen Kumar Suraneni, Alexandre Gaspar-Maia, Irawati Kandela, Pieter Van Vlierberghe, John D. Crispino, Aristotelis Tsirigos, Panagiotis Ntziachristos

**Affiliations:** ^1^ Department of Biochemistry and Molecular Genetics, Northwestern University, Chicago, IL, USA.; ^2^ Simpson Querrey Center for Epigenetics, Feinberg School of Medicine, Northwestern University, Chicago, IL, USA.; ^3^ Department of Biomolecular Medicine, Ghent University, Ghent, Belgium.; ^4^ Center for Medical Genetics, Ghent University and University Hospital, Ghent, Belgium.; ^5^ Cancer Research Institute Ghent (CRIG), Ghent, Belgium.; ^6^ Department of Pathology, New York University School of Medicine, New York, NY, USA.; ^7^ Laura and Isaac Perlmutter Cancer Center, New York University School of Medicine, New York, NY, USA.; ^8^ Applied Bioinformatics Laboratories, Office of Science and Research, New York University School of Medicine, New York, NY, USA.; ^9^ Division of Experimental Hematology, St. Jude Children’s Research Hospital, Memphis, TN, USA.; ^10^ Proteomics Center of Excellence, Northwestern University, Evanston, IL, USA.; ^11^ Oncohematology Laboratory, Department of Women’s and Children’s Health, University of Padova, Padova, Italy.; ^12^ Department of Surgery Oncology and Gastroenterology, Oncology and Immunology Section, University of Padova, Padova, Italy.; ^13^ Italian Institute for Genomic Medicine, Torino 10060, Italy.; ^14^ School of Pharmacy, University of Nottingham, Nottingham NG7 2RD, UK.; ^15^ Division of Experimental Pathology and Laboratory Medicine, Department of Laboratory Medicine and Pathology, Mayo Clinic, Rochester, MN, USA.; ^16^ University Children’s Hospital, Division of Pediatric Oncology, University of Zurich, Zurich, Switzerland.; ^17^ Department of Cell and Developmental Biology, Northwestern University, Chicago, IL, USA.; ^18^ Center for Developmental Therapeutics, Northwestern University, Evanston, IL, USA.

## Abstract

Dysregulation of kinase signaling pathways favors tumor cell survival and therapy resistance in cancer. Here, we reveal a posttranslational regulation of kinase signaling and nuclear receptor activity via deubiquitination in T cell acute lymphoblastic leukemia (T-ALL). We observed that the ubiquitin-specific protease 11 (USP11) is highly expressed and associates with poor prognosis in T-ALL. *USP11* ablation inhibits leukemia progression in vivo, sparing normal hematopoiesis. USP11 forms a complex with USP7 to deubiquitinate the oncogenic lymphocyte cell–specific protein-tyrosine kinase (LCK) and enhance its activity. Impairment of LCK activity leads to increased glucocorticoid receptor (GR) expression and glucocorticoids sensitivity. Genetic knockout of *USP7* improved the antileukemic efficacy of glucocorticoids in vivo. The transcriptional activation of GR target genes is orchestrated by the deubiquitinase activity and mediated via an increase in enhancer-promoter interaction intensity. Our data unveil how dysregulated deubiquitination controls leukemia survival and drug resistance, suggesting previously unidentified therapeutic combinations toward targeting leukemia.

## Teaser

The deubiquitinases USP7 and USP11 enhance LCK activity and hamper response to glucocorticoids in T-ALL.

## INTRODUCTION

Overcoming resistance to systemic therapy is an unmet need in acute leukemia (*1*–*7*). Glucocorticoids (GCs), such as dexamethasone (DXM), are essential components of therapeutic regimens for T cell acute lymphoblastic leukemia (T-ALL), and sensitivity to these drugs is the strongest predictor of treatment outcome (*8*). Mechanisms of resistance to therapy have been attributed to genetic variants (*9*–*12*) but can only explain 15 to 20% of relapsed leukemia cases.

Dysregulation of deubiquitination is involved in many disease states, including immune diseases, neurodegenerative disease, and cancer (*13*–*20*). In certain contexts of T cell leukemia and multiple myeloma, ubiquitin-specific protease 7 (USP7) drives the oncogenic programs (*21*–*24*). These findings underline the critical function of USPs in hematological malignancies. Lymphocyte cell–specific protein-tyrosine kinase (LCK) activity is required for proliferation and survival of T-ALL, and recently, it was demonstrated that a big proportion of pediatric and adult T-ALL cases respond to dasatinib, a well-known LCK inhibitor (*25*, *26*). Because LCK is known to be deubiquitinated in vivo, it is possible that USPs act on LCK as part of their role in promoting tumor survival (*27*). Furthermore, it has been shown that inhibition of LCK enhances the response to GCs in lymphoid malignancies (*28*–*31*). However, the mechanisms that regulate this response are not well understood.

We performed an unbiased analysis of deubiquitinase levels and association with disease prognosis, coupled to biochemical, molecular, and in vivo disease modeling studies. We demonstrate that USP11 plays an oncogenic role in lymphoid malignancy and controls LCK activity in association with USP7 via LCK deubiquitination. Impairment of LCK activity impedes T cell signaling, leading to up-regulation of GC receptor (GR) expression, which sensitizes the leukemia cells to GCs. In addition, USP7 inhibition enhances transcription activation of GCs target genes, such as *BCL2L11* and *TSC22D3*, through increasing the promoter and intronic GR-binding region (IGR) connectivity. These findings provide a rationale for the use of deubiquitinase or LCK inhibitors and GCs as combination treatments in leukemia.

## RESULTS

### USP11 is essential for T-ALL

**Fig. 1.**
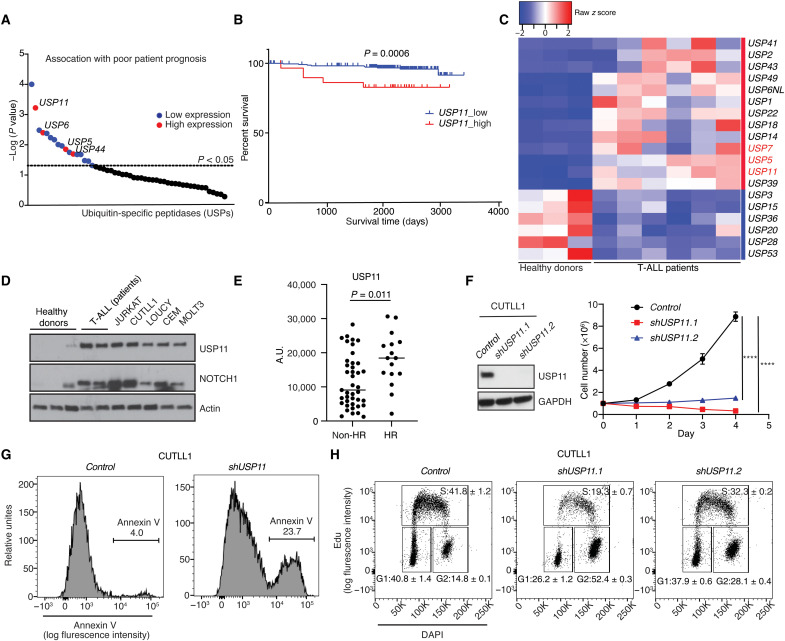
USP11 is essential for T cell leukemia. (**A**) *P* value of survival analysis of 52 USP-encoding transcripts in T-ALL patients based on their expression from the Pediatric Cancer Genome Project data portal (PeCan, St. Jude, Memphis). Red indicates high expression (top, 33%) that is associated significantly with poor prognosis, and blue indicates low expression (bottom, 66%) that is significantly associated with poor prognosis. (**B**) Survival of pediatric T-ALL patients based on low (blue line) or high (red line) *USP11* expression (source: PeCan). (**C**) Heatmap showing the expression of significant USPs in T-ALL patients (*n* = 6) and control T cells (CD3^+^ T cells, *n* = 3). (**D**) Western blot analysis for USP11 (top) and NOTCH1 (center) in control CD3^+^ T cells from the peripheral blood of healthy donors, bone marrow biopsies from T-ALL patients, and the human T-ALL cell lines JURKAT, CUTLL1, LOUCY, CEM, and MOLT13. (**E**) Reverse-phase protein array (RPPA) analysis of USP11 in non–high-risk [standard risk (*n* = 13) and medium risk (*n* = 28) combined] and high-risk (*n* = 16) groups of T-ALL patients (Mann-Whitney *t* test, *P* = 0.011). The risk group is classified by minimal residual disease (MRD) status (see Methods). A.U., arbitrary units; HR, high risk. (**F**) Immunoblot detection of USP11 protein levels (left) and growth curves of *control-* and *shUSP11*-expressing CUTLL1 cells over a period of 4 days (*n* = 3, right; ****P < 0.0001). Glyceraldehyde-3-phosphate dehydrogenase (GAPDH) is used as loading control. (**G**) Annexin V staining (72 hours) of CUTLL1 cells that expressed either *control* shRNA or *shUSP11*. Experiment was repeated three times, and a representative example is shown. (**H**) Edu staining (72 hours) following *control* shRNA or *shUSP11* in CUTLL1 cells. The means ± SD from two representative studies are shown.

Previous studies have demonstrated important oncogenic roles for the process of deubiquitination, including stabilization of Neurogenic locus notch homolog protein 1 (Notch1) and serine/arginine-rich splicing factor 6 (SRSF6) by the deubiquitinase USP7 in T-ALL (*21*, *22*). To further characterize the oncogenic role of USPs in T-ALL, we analyzed survival data from the pediatric cancer genome project (PeCan) data portal in association with the expression levels of 52 USP family members. We identified four USPs whose high transcript expression associates with poor prognosis: *USP11*, *USP6*, *USP5*, and *USP44* (Fig. 1A). *USP11* presents the lowest *P* value among those USPs (Fig. 1B and fig. S1A). *USP5* has been shown to play oncogenic roles in solid tumors (*32*). USP11, which is encoded by the X-linked *USP11* gene, might exist in a complex with USP7 and is a key modulator of cell cycle progression, protein synthesis, and DNA damage repair (*33*–*36*). USP11 stabilizes and enhances Eukayotic translation initiation factor 4B (eIF4B) activity and promotes oncogenic translation in diffuse large B cell lymphoma (DLBCL) (*34*). Because of the involvement of USP11 in DLBCL and its presumed interaction with USP7, we analyzed gene expression using a separate cohort of pediatric T-ALL samples to reveal an increased *USP11 *expression compared to healthy T cells (Fig. 1C and fig. S1B). Similarly, we identified elevated *USP11* transcript expression in patient samples compared to thymocytes (fig. S1C). Additional analysis demonstrated that USP11 protein levels were higher in T-ALL patients and cell lines compared to normal T cells (Fig. 1D). Reverse-phase protein array (RPPA) experiments showed a higher expression of USP11 protein in high-risk (refractory or relapsed) patients compared to the non–high-risk group (Fig. 1E). Our data demonstrate that USP11 is highly expressed in T-ALL cases with poor prognosis.

### Targeting *USP11* impedes T-ALL growth, sparing normal hematopoiesis and thymus development

Next, we assessed the importance of USP11 in T-ALL. RNA interference–based silencing *USP11* using two short-hairpin RNAs (shRNAs), *shUSP11.1* and *shUSP11.2*, leads to inhibition of cell growth (Fig. 1F and fig. S2, A and B) due to an increase in apoptosis and a cell cycle arrest at the S phase (Fig. 1, G and H, and fig. S2, C and D). Moreover, a peptide ligand, USP11-FYLIR, which serves as a protein-protein interaction inhibitor (*37*), demonstrates a similar inhibition of cell growth (fig. S2E).

**Fig. 2.**
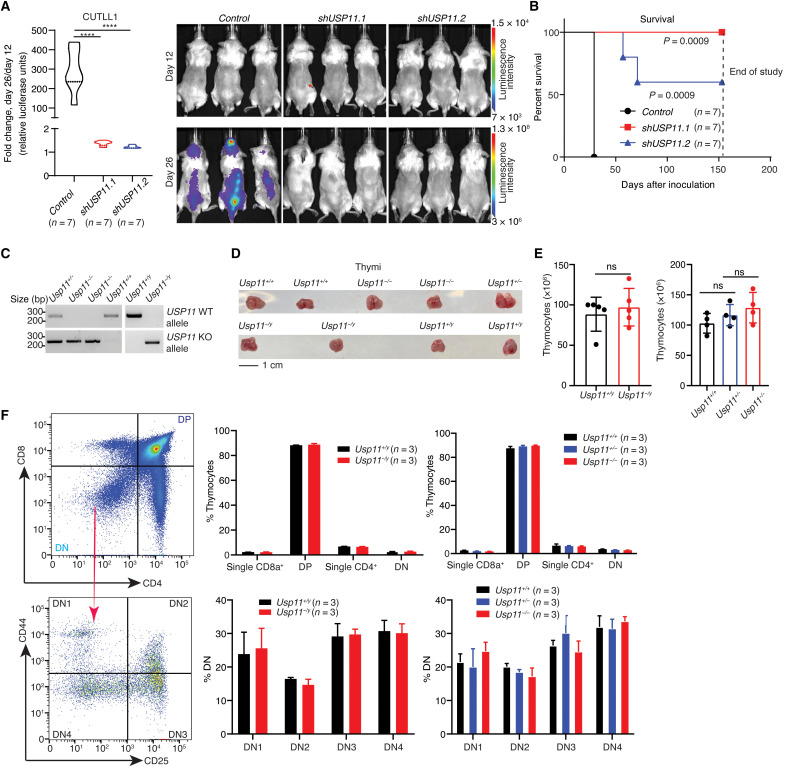
In vivo targeting of *USP11* impedes T cell leukemia growth, sparing thymic development. (**A**) Quantification of tumor growth in vivo (left) and representative bioluminescence pictures of immunocompromised animals (right) that were transplanted (intravenous injection) with *Luciferase*-expressing CUTLL1 cells that were previously transduced with a lentiviral vector expressing either a control hairpin RNA, *shUSP11.1*, or *shUSP11.2*, and selected using puromycin for a period of 3 days. Leukemic burden was assessed twice per week by bioluminescence measurements. Relative bioluminescence intensity is shown for three representative mice per group on days 12 and 26 after transplantation (right). The fold change in total flux from days 12 to 26 is shown on the left (****P < 0.0001). (**B**) Survival analysis of immunocompromised mice transplanted with control hairpin RNA-, *shUSP11.1-*, or *shUSP11.2*-expressing CUTLL1 cells. *P* value was calculated by log-rank (Mantel-Cox) test. (**C**) Genotype confirmation of wild-type (WT) or knockout (KO) mice for the detection of the *Usp11* allele in tail DNA. (**D**) Representative thymi in different *Usp11* genotypic mice. (**E**) Total number of thymocytes in *Usp11^+/y^* (*n* = 5), *Usp11^−/y^* (*n* = 5), *Usp11^+/+^* (*n* = 4), *Usp11^+/−^* (*n* = 4), and *Usp11^−/−^* (*n* = 4) mice at age 6 to 8 weeks. ns, not significant. (**F**) Flow cytometry analysis of major thymic subsets in mice with different *Usp11* alleles is shown. Representative images of the flow cytometry analysis are shown on the left. Relative proportions of the major cell populations in the thymus of *Usp11^+/y^* (*n* = 3), *Usp11^−/y^* (*n* = 3), *Usp11^+/+^* (*n* = 3), *Usp11^+/−^* (*n* = 3), and *Usp11^−/−^* (*n* = 3) mice is shown in the bar graphs on the right. DP cells express both CD4 and CD8 markers (“double positive”), whereas the DN (double negative) population are negative for both CD4 and CD8, which can be further classified as DN1 (CD44^+^/CD25^−^), DN2 (CD44^+^/CD25^+^), DN3 (CD44^−^/CD25^+^), and DN4 (CD44^−^/CD25^−^). ns, not significant.

To further characterize the essential role of USP11 in T-ALL progression in vivo, we used a xenograft model. *shUSP11*-expressing T-ALL cells were transplanted intravenously into immunocompromised mice, and tumor growth in vivo was monitored. *USP11* silencing showed a notable inhibition of tumor growth (Fig. 2A and fig. S3A) and enhanced mouse survival (Fig. 2B and fig. S3B). We further used doxycycline-inducible *shUSP11* expression (*shUSP11.a*) after disease establishment (fig. S3, C and D). USP11 ablation inhibited tumor growth, suggesting a critical role for USP11 in T-ALL maintenance (fig. S3, E and F). These data demonstrate that USP11 ablation suppresses T-ALL growth in vivo.

To investigate whether ablation of the *Usp11* gene affects normal hematopoiesis and thymic development, we used a germline *Usp11* knockout mouse (Fig. 2C and fig. S3, G and H). Littermate offspring numbers follow the Mendelian proportions, and the mice do not present any significant physiological defects, suggesting that *Usp11* is dispensable for mouse development [ (*38*); see also fig. S3H]. Examination of *Usp11^−/−^* and *Usp11^−/y^* mice showed no significant changes either in peripheral blood populations over a period of 6 months (fig. S3I) or in the size or cellularity of the thymus (Fig. 2, D and E). Furthermore, thymocyte development is largely unaffected in the absence of *Usp11* (Fig. 2F). These results suggest a potential therapeutic window for targeting USP11 in leukemia.

### USP11 and USP7 form an oncogenic complex with LCK in T-ALL

**Fig. 3.**
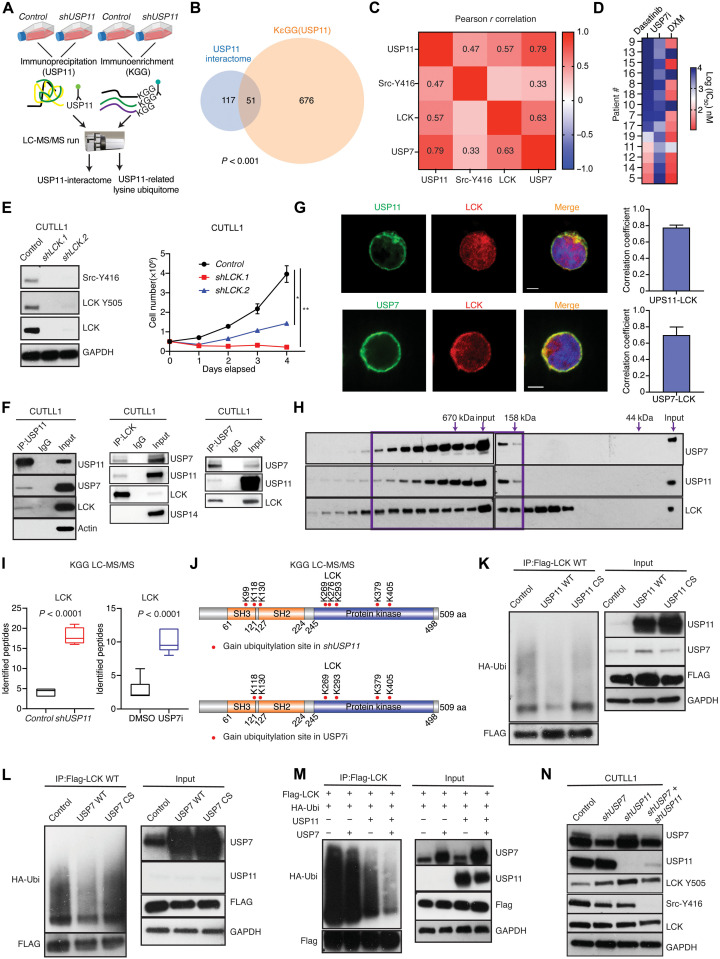
USP11 and USP7 form an oncogenic complex with LCK and control its activity through deubiquitination in T cell leukemia. (**A**) Schematic representation of USP11-related immunoprecipitation mass spectrometry (MS) and lysine ubiquitome analysis in CUTTL1 cells. (**B**) Analysis of the overlapping datasets for USP11 immunoprecipitation and KεGG MS studies. (**C**) Heatmap representation of correlation for USP7, USP11, Src-Y416, and LCK in T-ALL patients by RPPA analysis (significant Pearson *r* values were shown, *P* < 0.05). (**D**) Heatmap showing concentrations causing 50% cell growth inhibition (GI50) for DXM, dasatanib, and USP7 inhibitor. (**E**) Immunoblot detection of LCK, LCK phospho-Y505, Src phospho-Y416, and GAPDH (left). Growth of *control*- and *shLCK*-expressing CUTLL1 cells (right; *n* = 3; *P < 0.05 and **P < 0.01). (**F**) Immunoblot studies following immunoprecipitation (IP) for USP11 (left), LCK (middle), and USP7 (right) in CUTLL1 cells. USP7, USP11, LCK, USP14, and actin were detected. (**G**) Colocalization of USP11 (green) and UPS7 (green) with LCK (red) in CUTLL1 cells. Scale bars, 5 μm. Pearson’s correlation score of internalized USP11 and LCK (top right) or USP7 and LCK (bottom right). (**H**) Immunoblot studies for LCK, USP7, and USP11 following isolation of whole-cell extracts and gel filtration chromatography in JURKAT cells. (**I**) Box plot showing ubiquitinated peptides of LCK upon *shUSP11* (left) and USP7i treatment (10 μM) (right). (**J**) Analysis of gained lysine ubiquitination sites within LCK upon *shUSP11* (top) and USP7i treatment (bottom). (**K**) Ubiquitination assay for LCK ubiquitination status in 293 T cells in the presence of WT or mutant (CS) *USP11*. (**L**) Ubiquitination assay for LCK ubiquitination status in 293 T cells in the presence of WT or mutant (CS) *USP7*. (**M**) Ubiquitination analysis of LCK in 293 T cells overexpressing USP7, USP11, or both. (**N**) Immunoblot detection of USP11, USP7, LCK, LCK phospho-Y505, Scr phospho-Y416, and GAPDH in CUTLL1 cells expressing control *shRNA*, *shUSP7*, *shUSP11*, and *shUSP7* plus *shUSP11*.

Next, we wanted to identify direct USP11 substrates (Fig. 3A). USP11 immunoprecipitation coupled to mass spectrometry (MS) analysis identified 168 interactors (table S1), including the oncogenic deubiquitinase USP7 (*21*, *22*). Previous studies have identified USP11 as an USP7 interactor (*21*, *36*, *39*, *40*). Assessment of USP7 protein levels in a patient cohort showed that the USP7 protein is highly expressed in high-risk disease (fig. S4A). Overlap of the USP11 interactome dataset with differentially ubiquitinated proteins after *USP11* silencing (727 proteins; table S2) identified 51 proteins as direct USP11 substrates (Fig. 3B). Inhibition of USP7 and USP11 silencing led to a substantial overlap of ubiquitination [Ubiquitin Remnant Motif, Lys-ϵ-Gly-Gly (KεGG)] signatures, including genes associated with the T cell receptor (TCR) pathway (fig. S4B). Further essentiality analysis of the USP11 substrates demonstrated that LCK is an essential gene specifically in T cell leukemia among 49 cancer types (fig. S4, C and D).

LCK has been shown to play a critical role in the T cell development and activation through regulation of TCR signaling (*41*, *42*). Expression analysis for *USP7*, *USP11*, and *LCK* mRNA levels showed that they are substantially expressed in all T-ALL subgroups (fig. S4, E to G). USP7, USP11, Src-Y416, and LCK protein levels showed a positive correlation in T-ALL patients (Fig. 3C). Total LCK and the phosphorylated active LCK are highly expressed in T-ALL compared to normal T cells (fig. S5B), suggesting LCK hyperactivation in disease. Treatment of a panel of patient samples using USP7i and the LCK inhibitor dasatinib showed that some T-ALLs are sensitive to these compounds, whereas no differences were observed on the levels of USP7, USP11, and Src-Y416 in dasatinib-sensitive or dasatinib-insensitive group (fig. S5A). *LCK* silencing led to increased apoptosis and impaired cell growth (Fig. 3E and fig. S5, C and D), similar to chemical inhibition of LCK (using dasatinib, bosutinib, and WH-4-023; fig. S5, E to G). Ectopic LCK expression partially rescued *shUSP11*-inflicted inhibition of T-ALL cell growth, suggesting that LCK is a critical USP11 substrate (fig. S5H).

We hypothesized that USP11 and USP7 form an oncogenic complex with LCK. The interaction between USP11/USP7 and LCK was verified by using immunoprecipitation studies (Fig. 3F and fig. S6, A to D), also supported by immunofluorescence staining showing colocalization (Fig. 3G). Gel filtration chromatography suggested that USP7, USP11, and LCK were in the same complex (Fig. 3H). Of note, we did not detect interaction between LCK and USP7 or USP11 in unstimulated mouse thymocytes (fig. S6E). Together, these findings demonstrate that USP11 and USP7 form an oncogenic complex with LCK in leukemia.

### LCK deubiquitination controls its activity

To test whether USP11 and USP7 regulate LCK deubiquitination, our KεGG-MS analysis revealed that the total numbers of ubiquitinated peptides of LCK were increased upon *shUSP11* expression or USP7 inhibition (Fig. 3I). We identified eight lysine sites in LCK with increased ubiquitination upon *shUSP11* treatment and six sites with increased ubiquitination upon treatment with USP7i (Fig. 3J and fig. S6F). All altered lysines were in the Src homology 2 (SH2), SH3, and the kinase domains of LCK, and notably, all six differentially ubiquitinated lysines in USP7i were observed in *shUSP11* as well (K118, K130, K269, K293, K379, and K405), indicating that USP7 and USP11 might cooperate in regulating LCK.

To confirm the deubiquitination of LCK by USP7 and USP11, ectopic expression of catalytically active USP11, but not the inactive mutant of USP11 and USP11^C275S^, reduced the overall ubiquitination of LCK in 293 T cells (Fig. 3K). Similar results for LCK ubiquitination were obtained by using USP7 and the catalytically inactive mutant USP7^C223S^ (Fig. 3L). Furthermore, the effect on deubiquitination of LCK was stronger upon co-overexpression of USP7 and USP11 compared with overexpression of USP7 or USP11 alone, further confirming that USP7 and USP11 cooperate to deubiquitinate LCK (Fig. 3M). Next, we investigated the type of ubiquitination regulated by USP11 on LCK. Deubiquitination analysis showed that USP11 deubiquitinates LCK when K27, K29, or K33-only ubiquitin was presented (fig. S6G). These results demonstrate that the USP11/USP7 complex regulates the deubiquitination of LCK.

We then characterize how deubiquitination might regulate LCK. Previous analysis of sites associated with proteasomal degradation or activity of kinases (*43*) suggests that ubiquitination of four of the common lysines (K130, K269, K293, and K379) is not associated with proteasomal degradation (“proteasome-insensitive”). This finding suggests that LCK ubiquitination might control its activity rather than its levels. Notably, silencing of *USP11* in T-ALL cells led to an increase of the inactive LCK phospho-Y505 and a decrease of the active LCK phospho-Y394 without affecting the expression of total LCK protein (fig. S6H). Of note, inhibition of USP7 or combined attenuation of USP7 and USP11 levels also affected phosphorylated LCK levels (Fig. 3N and fig. S6I). These results illustrate that USP11 and USP7 cooperatively control LCK activity.

### Deubiquitination controls LCK signaling and GC signaling

Given the essential role of LCK and its activity in T-ALL maintenance, we investigate the downstream effect of LCK deubiquitination in T-ALL. Global protein levels and protein phosphorylation levels upon *USP11* silencing were performed (tables S3 to S5). We observed 356 proteins with lower expression levels [down (DN)] and 99 proteins with higher expression levels (UP) upon USP11 silencing (fig. S7A). Like *shUSP11*, USP7i also presents a reduction in protein levels (140 DN and 52 UP proteins; fig. S7B). Dasatinib treatment, in contrast, led to very limited changes in protein expression (three DN and five UP proteins) (fig. S7C). Of note, attenuation of USP7 or USP11 leads to similar global changes in protein levels (fig. S7D). In line with the previously suggested role of USP11 in DNA damage repair (*35*, *44*, *45*) and G_2_-M cell cycle arrest upon knockdown of *USP11* (Fig. 1H), gene ontology analysis of USP11–down-regulated proteins showed an enrichment for cell cycle regulation and DNA repair pathways (fig. S7E). Increased levels of proteins participating in catabolic processes were detected upon *USP11* silencing, in line with previous findings suggesting that USP11 favors anabolic processes (fig. S7F) (*34*).

**Fig. 4.**
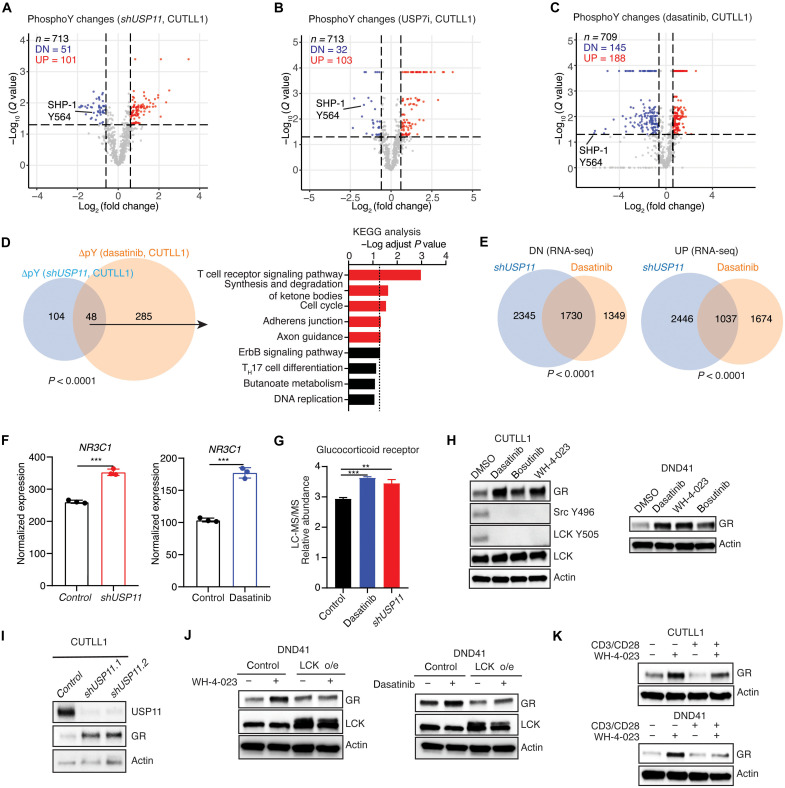
Disruption of LCK activity dampens TCR signaling and increases GR expression. (**A** to **C**) Volcano plots showing changes in peptides with phospho-tyrosines in *shUSP11* versus control, USP7i (5 μM) versus control, and dasatinib (5 μM) versus control comparisons in CUTLL1 cells. Multiple unpaired *t* test analyses (*P* value) followed by false discovery rate (FDR) (*Q* value) analysis were performed. UP, increase; DN, decrease. Y564 phosphorylation of the well-characterized LCK substrate SHP-1 is shown. (**D**) Venn diagram showing overlap of peptides with phospho-tyrosines in *shUSP11* versus control and dasatinib versus control comparisons in CUTLL1 cells (left). KEGG analysis for overlapping proteins (right). (**E**) Analysis of RNA-seq data showing the overlap of down-regulated genes (DN, left) or up-regulated genes (UP, right) upon dasatinib (5 μM) treatment or *sh*USP11** in CUTLL1 cells. (**F**) Analysis of RNA-seq data showing the expression of *NR3C1* in control and *shUSP11* groups (left or control and dasatinib treatment groups (right) (*n* = 3, ***P < 0.001). (**G**) Analysis of quantitative proteomics data showing the expression of GR in control (*n* = 3), dasatinib-treated (*n* = 2, ***P < 0.001), and *shUSP11* cells (*n* = 3, **P < 0.01). (**H**) Immunoblot detection of GR, LCK, LCK phospho-Y505, Scr phospho-Y416, and actin upon treatment with dasatinib (5 μM), bosutinib (5 μM), and WH-4-023 (5 μM) for 6 hours in CUTLL1 cells (left) or DND41 cells (GR detection is shown, right). DMSO, dimethyl sulfoxide. (**I**) Immunoblot detection of USP11, GR, and actin in *control* and *shUSP11*-expressing CUTLL1 cells. (**J**) Immunoblot detection of LCK, GR, and actin upon ectopic expression of LCK (LCK o/e) in DND41 cells coupled to treatment with WH-4-023 (2 μM) or dasatinib (2 μM) for 6 hours. (**K**) Immunoblot detection of GR and actin upon treatment of CUTLL1 cells (top) or DND41 cells (bottom) with CD3/CD28 beads or WH-04-23 (2 μM) for 6 hours.

Then, USP11-related global phosphorylation changes were assessed. A total of 152 tyrosine phosphopeptides (Q<) presented changes; 51 of them showed a decrease and 101 had increased phosphorylation (Fig. 4A). USP7i led to 32 peptides with decreased tyrosine phosphorylation and 103 peptides with increased phosphorylation (Fig. 4B). Dasatinib treatment for LCK inhibition, in turn, yielded 145 peptides with decreased and 188 peptides with increased tyrosine phosphorylation (Fig. 4C). TCR signaling proteins exhibited phosphorylation pattern changes upon all three conditions (fig. S7, G to I). A notable overlap of the phosphorylation pattern changes between dasatinib treatment and *USP11 *silencing or USP7i was observed, and TCR proteins were enriched, suggesting that LCK activity is critical for TCR signaling (Fig. 4D and fig. S7J). Similarly, pY564 levels of the LCK substrate tyrosine-protein phosphatase non-receptor type 6 (PTPN6; also known as SHP1) (*46*) decreased significantly in all three treatment conditions similarly to the pY319 levels on ZAP70, an LCK substrate (Fig. 4, A to C, and fig. S7, K and L) (*47*). The total ZAP70 and SHP1 levels remained unchanged. Our findings demonstrate that USP11 and USP7 control the activity and downstream substrates of LCK.

To further investigate the role of LCK inhibition in gene expression, RNA sequencing (RNA-seq) was performed upon dasatinib treatment or *USP11 *silencing. Notably, these datasets exhibit significant overlap with each other on increased or decreased transcripts (Fig. 4E). As LCK activity is associated with GC response, we scanned for changes in the GR pathway upon *shUSP11* and found that *NR3C1* is up-regulated (Fig. 4F) (*28*, *31*). In line with this finding, our proteomics analysis upon *shUSP11* or dasatinib treatment showed an increase of GR proteins levels (Fig. 4G). Moreover, *USP11* or *LCK* knockdown, USP7 inhibition, and LCK inhibition led to increased GR levels (Fig. 4, H and I, and fig. S8, A and B), and this increase was abrogated when LCK was overexpressed in the system (Fig. 4J). Similar results were obtained upon TCR stimulation (Fig. 4K and fig. S8, C and D). Thus, LCK impairment leads to activation of *NR3C1*/GR expression through TCR signaling.

### USP7/USP11-LCK complex controls response to GCs via up-regulation of GR expression in T-ALL

**Fig. 5.**
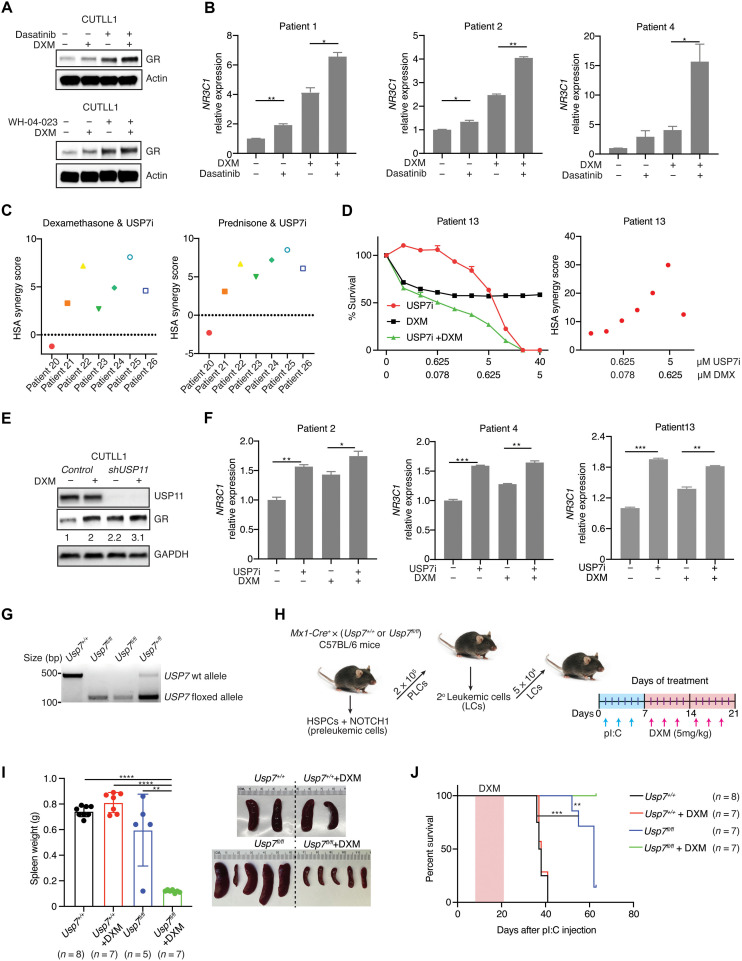
Impairment of the LCK-USP7/USP11 axis enhances GC response in T cell leukemia in vitro and in vivo. (**A**) Immunoblot detection of GR and actin upon DXM (1 μM), dasatinib (2 μM), or combination treatment for 6 hours (top) and DXM (1 μM), WH-04-23 (2 μM), or combination treatment for 6 hours (bottom) in CUTLL1 cells. (**B**) Reverse transcription (RT)–quantitative polymerase chain reaction (qPCR) analysis of *NR3C1* in T-ALL patient samples treated with DMSO, DXM (100 nM), dasatinib (2 μM), or the combination for 6 hours (*P < 0.05 and **P < 0.01). (**C**) Highest Single Agent (HSA) synergy score for USP7 inhibitor and DXM (left) or USP7 inhibitor and prednisone (right) for 5 days in seven patient samples. (**D**) Dose response curves from DXM, USP7i, and the combination treatment (left) and HSA synergy score (right). The error bars represent the SD of two technical replicates. (**E**) Immunoblot detection of GR and GAPDH with or without DXM treatment (1 μM) in *shUSP11-*expressing CUTLL1 cells. Relative GR expression is normalized to GAPDH. (**F**) RT-qPCR analysis of *NR3C1* in patient samples treated with DXM [20 nM (patients 2 and 4) or 50 nM (patient 13)], USP7i (5 μM), or their combination for 6 hours (*P < 0.05, **P < 0.01, and ***P < 0.001). (**G**) Genotype confirmation of the floxed allele of *Usp7* in tail DNA. (**H**) Schematic representation for generating NOTCH1-driven leukemic cells from *Mx1Cre^+^*-*Usp7^fl/fl^* or *Mx1Cre^+^*-*Usp7^+/+^* mice. The leukemic cells (LCs) were transplanted in secondary recipient mice, treated with vehicle (phosphate-buffered saline) or with DXM (5 mg/kg). HSPCs, hematopoietic stem progenitor cells; PLCs, preleukemia cells; pI:C, polyinosinic:polycytidylic acid. (**I**) Spleen weight from secondary recipient mice when humane end points were reached or on day 63 (end of the study, left; **P < 0.01 and ****P < 0.0001). Representative images of spleens (right). (**J**) Survival curve of the recipient mice from the secondary transplants in (I). *P* value was calculated by log-rank (Mantel-Cox) test (**P < 0.01 and ***P < 0.001).

LCK activity controls the response to GCs in lymphoid tumors (*28*–*31*, *48*, *49*). Thus, CUTLL1 cells were treated with DXM to evaluate the effect of GCs on the USP7/USP11-LCK complex. We observed a decreased LCK-USP7/USP11 interaction (fig. S9A) and decreased LCK phosphorylation (fig. S9B) upon DXM treatment in T-ALL cells. However, no direct interaction between LCK and GR was observed (fig. S9, C and D). These results suggest that DXM and LCK signaling pathways cross-talk and up-regulation of GR caused by impairment of LCK might increase GC efficacy to induce apoptosis. Dasatinib and DXM showed a synergistic effect in T-ALL cells (fig. S9, E to G). Moreover, we observed increased GR expression upon combination of LCK inhibitors and DXM treatment, suggesting that the synergistic effect between DXM and LCK inhibition in T-ALL results from GR up-regulation (Fig. 5A and fig. S9H). This *NR3C1* up-regulation was confirmed in eight of the eight primary T-ALL patient samples in the dasatinib treatment group with or without DXM (Fig. 5B and fig. S9, I and J). Similar results were found when treated with WH-04-023, another LCK inhibitor, in the presence of DXM (fig. S9K). Of note, no *NR3C1* expression difference was observed upon LCK inhibition in unstimulated human CD3^+^ cells (fig. S9L).

Modulating LCK activity via *USP11* silencing or USP7 inhibition sensitized T-ALL cells to the GC treatment, as evidenced by inhibition of cell growth caused by an increase in apoptosis upon GC treatment (fig. S10, A to H). Notably, USP7 inhibitor and DXM or prednisone showed various synergistic effects in inhibiting T-ALL growth, ranging from mild to strong, in seven of the eight primary T-ALL patient samples tested (Fig. 5, C and D, and figs. S11 and S12). Furthermore, an increase of GR expression was observed upon GC treatment in the *shUSP11* group (Fig. 5E). USP7 inhibition or knockdown leads to increase of *NR3C1* upon GC treatment in T-ALL cell lines (fig. S10, I and J) and patient samples (Fig. 5F). We demonstrated that disruption of the USP7/USP11-LCK complex sensitizes T-ALL to GC treatment via *NR3C1* up-regulation.

### Genetic knockout of USP7 sensitizes T-ALL to GCs in vivo

To further evaluate the role of USP7 in resistance to GCs in vivo, we used a characterized conditional *Usp7* knockout mouse model (*Mx1Cre-Usp7^−/−^*; Fig. 5G) (*50*). *Mx1Cre-Usp7^fl/fl^* and *Mx1Cre-Usp7^+/+^* mice were used to develop a NOTCH1-induced in vivo T-ALL mouse model that was treated with vehicle or DXM (Fig. 5H). *Usp7* knockout relieves leukemia burden as demonstrated by reduced spleen weight coupled to prolonged survival, an effect that is further enhanced upon mouse treatment with DXM (Fig. 5, I and J). This suggests that genetic ablation of USP7 can sensitize T-ALL cells to DXM treatment.

### Chromatin accessibility, organization, and gene expression regulation by USPs in the context of GCs

**Fig. 6.**
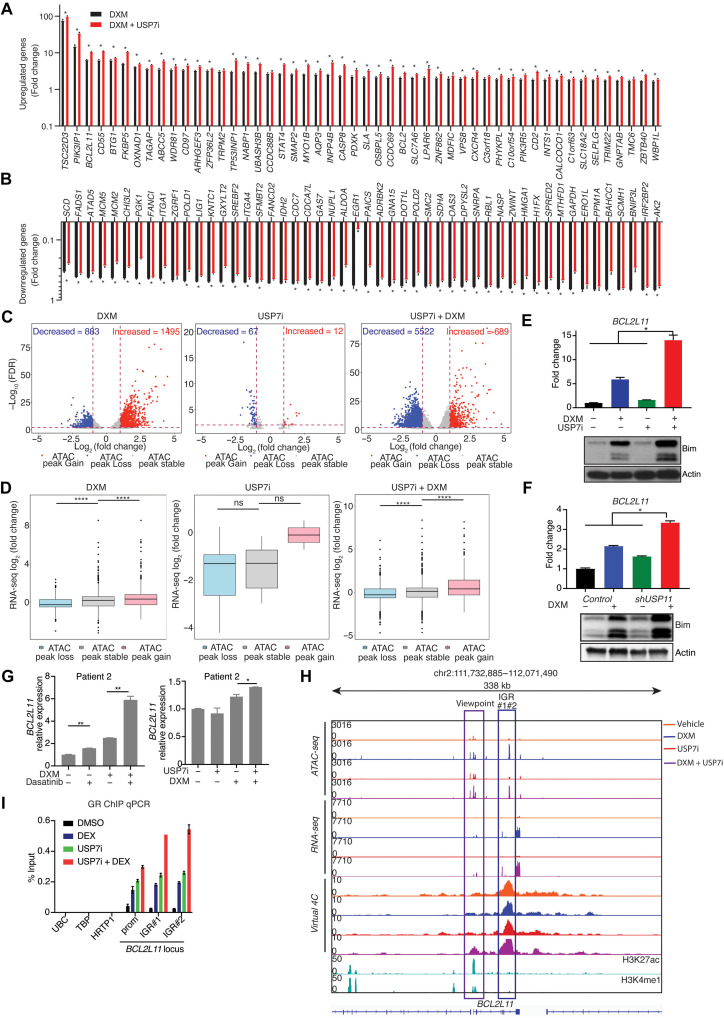
USP7 inhibition potentiates GC-induced chromatin organization changes and transcriptional activation in proapoptotic loci. (**A** and **B**), Gene expression analysis using RNA-seq (*n* = 3, *P < 0.05). The top 50 genes activated (A) and repressed (B) by DXM and combination (USP7i + DXM)–treated DND41 cells. (**C**) Volcano plot showing ATAC peak changes in the DXM group (left), USP7i group (middle), or combination group (right), normalized to the control group in DND41 cells. (**D**) Gene expression analysis for transcripts associated with loss, stable, or gained ATAC signal for the same groups shown in (C) (****P < 0.0001). (**E**) RT-qPCR analysis of *BCL2L11* in DND41 cells treated with DXM (1 μM), USP7i (5 μM), or their combination for 24 hours (top). Immunoblot detection of BIM and actin in DND41 cells (bottom, *P < 0.05). (**F**) *BCL2L11* expression using RNA-seq analysis performed upon *shUSP11* in CUTLL1 cells treated with DXM or not (top). Immunoblot detection of BIM and actin is shown (bottom) in DND41 cells (*P < 0.05). (**G**) RT-qPCR analysis of *BCL2L11* in T-ALL patient sample treated with DMSO, DXM (100 nM), dasatinib (2 μM), or their combination for 6 hours (**P < 0.01) (left) or DMSO, DXM (20 nM), USP7i (5 μM), or their combination for 6 hours (*P < 0.05) (right). (**H**) Snapshots of *BCL2L11* loci (IGV browser) presenting with gene expression (RNA-seq), chromatin accessibility (ATAC-seq), genomic interactions (virtual 4C), H3K27ac, and H3K4me1 status upon combination of GCs (DXM), USP7i, or their combination. Viewpoint, promoter of *BCL2L11* (purple square); IGR, intronic GR-binding region (blue square). (**I**) Chromatin immunoprecipitation (ChIP)–qPCR analysis of GR binding on the promoter (prom) or IGR1/2 of *BCL2L11* (*51*) or non–GR-bound genes *UBC*, *TBP*, and *HRTP1* in DND41 cells treated with of DXM (1 μM), USP7i (5 μM), or their combination for 6 hours. Data are plotted as percentage of the input DNA (1%). The error bars represent two technical replicates (*P < 0.05).

GCs induce GR translocation into the nucleus to increase or decrease target gene transcription. We found a marked enhancement of GC-induced changes in gene expression levels in the combination treatment group in comparison with single DXM or USP7i treatment group (Fig. 6, A and B).

To assay for chromatin changes in response to GC or USP7i treatment (*51*–*55*), we evaluated chromatin accessibility changes via Assay for Transposase-Accessible Chromatin using sequencing (ATAC-seq) following USP7 inhibition, DXM treatment, or their combination. Differential ATAC-seq peak analysis showed a marked decrease of ATAC peaks upon combination treatment compared with DXM treatment (5522 lost peaks in combination versus 883 lost peaks in DXM) (Fig. 6C, left and right), suggesting that USP7 inhibition leads to lower accessibility in the presence of GCs. USP7 inhibition alone, in contrast, causes minimal changes (Fig. 6C, middle). Of note, in USP7i, DXM, and combination treatment, most ATAC changes happened in distal intergenic loci (fig. S13A). Integration of RNA-seq and ATAC-seq analysis showed that a loss of chromatin accessibility associates with a decrease in gene expression, whereas increased chromatin accessibility associates with an increase in gene expression (Fig. 6D and fig. S13B). We identified that enrichment in the TCR and interleukin-4 (IL-4) pathways in the transcripts was negatively affected by DXM and that the negative regulation of the apoptotic process pathway was negatively affected in the combination treatment (fig. 13, C and D). On the other hand, genes associated with positive regulation of the apoptotic process, such as *BCL2L11* (*56*), were positively affected (fig. S13, B, E, and F).

*BCL2L11* was one of the top differentially expressed genes upon DXM treatment, and its transcript and protein levels were enhanced upon combination treatment (Fig. 6E). This is consistent with the enhanced effect of combination treatment on apoptosis (fig. S11, A to G). *USP11* silencing also led to increased Bcl-2-like protein 11 [Bcl-2 Interacting Mediator of cell death (BIM)] levels in the presence of GCs (Fig. 6F). This increase in GC-induced *BCL2L11* expression was validated using patient samples (Fig. 6G and fig. S14, A and B).

We then investigated the effect of GCs and USP7i on genome organization via chromatin conformation capture (Hi-C). Hi-C-bench analysis (*57*) showed that all three treatment categories showed a small number of chromatin A (active)/B (inactive) transitions (fig. S15A). Of note, the combination treatment showed the greatest number of B→A compartment shifts (fig. S15, B and C) and a decrease in intra-TAD (topologically associating domains) activity (fig. S15, D to F). Moreover, analysis of B→A compartment changes did not show any overlaps among the three treatment groups. Overlapping A→B compartment shift loci between DXM and combination treatment revealed shared differentially expressed genes that may not be directly associated with the proapoptotic phenotype. Of note, integration of ATAC-seq and intra-TAD activity analysis showed that loss of intra-TAD activity upon GC treatment associates with lower chromatin accessibility, whereas USP7 inhibition or combination treatment showed lower chromatin accessibility independently of intra-TAD activity (fig. S16A).

**Fig. 7.**
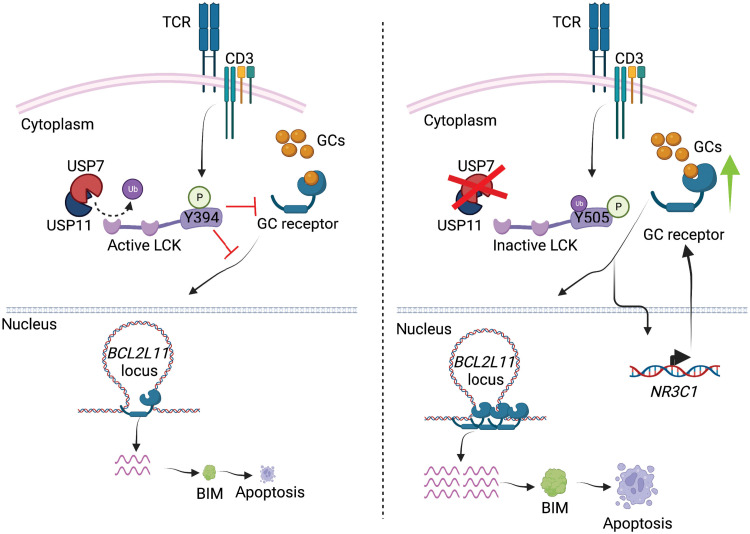
The role of LCK-USP7/USP11 axis and impact of its targeting on GC response. USP7 and USP11 deubiquitinate LCK to maintain the protein in the active, Y394-phosporylated state leading to inhibition of GR signaling pathway activity reduced GR binding to proapoptotic genes and reduced apoptosis upon application of GCs (left). We present that targeting USP7/USP11 (or LCK) leads to the up-regulation of *NR3C1* transcript and GR protein expression and concomitant enhancement of GR signaling pathway activity (green arrow), which induces apoptosis upon GC treatment via chromatin changes and increased expression of proapoptotic genes (right).

As general analysis of compartment and intra-TAD activity changes cannot provide sufficient evidence to explain the apoptosis-related phenotype, we focused on the *BCL2L11* loci. The *BCL2L11* promoter and an intragenic enhancer area that was previously characterized as IGR showed increased chromatin accessibility upon DXM and combination treatments but not USP7i treatment (Fig. 6H) (*51*). We also observed an increased binding of GR on *BCL2L11* promoter and IGR region upon combination treatment compared to treatment with DXM alone (Fig. 6I). Consistent with previous studies that the loop interaction between IGR and the promoter is critical for BIM activation, we show that USP7i and DXM treatment potentiates *BCL2L11* expression via enhancement of enhancer-promoter interaction compared with DXM (Fig. 6H). Analysis of another GR transcriptional target gene, *TSC22D3*, also showed an increasing connectivity between the promoter region and IGR upon DXM treatment and a further strengthening of this interaction upon combination treatment (fig. S16B). USP7i alone does not significantly affect gene expression, chromatin accessibility, or genomic connectivity in *BCL2L11* or *TSC22D3* loci (Fig. 6H and fig. S16B). Our overall findings demonstrate that targeting LCK/USP7/USP11 leads to down-regulation of TCR signaling, culminating in up-regulation of GR expression, which induces apoptosis via augmented GCs target expression upon GC treatment (Fig. 7).

## DISCUSSION

Dysregulation of deubiquitination as a modulator of oncogenic state in hematopoietic malignancies is largely uncharacterized. In this study, we have demonstrated a previously unidentified role for the deubiquitinase complex of USP7 and USP11 in regulating LCK kinase signaling and therapy response in T cell leukemia. USP-mediated deubiquitination maintains LCK in the active pY394 conformation that inhibits activation of apoptotic pathways upon application of GCs. LCK activity is mainly involved in regulation of TCR signaling (*42*). The thymocyte maturation process is regulated by LCK availability, and the deubiquitinating enzyme Cylindromatosis (CYLD) has been implicated in this process (*27*, *58*). However, CYLD expression is repressed in T-ALL patient samples (*59*, *60*), and our data demonstrate that LCK is differentially regulated in the thymus compared to leukemia settings. Of note, TCR stimulation resembles a molecular program similar to thymic negative selection showing antileukemic properties in T-ALL (*61*).

GCs are effective inhibitors of T cell immunity by causing impaired cytokine production and effector function (*62*, *63*). These processes are tightly intertwined with TCR signaling, as addition of GCs attenuates TCR signaling by causing dissociation and blocking phosphorylation of TCR-associated proteins (*49*, *64*). Moreover, inhibition of LCK activity sensitizes lymphoid leukemia cells to GCs, whereas its mechanism is largely uncharacterized (*28*, *29*, *31*). Our results suggest that inhibition of LCK activity by tyrosine kinase inhibitors or impairment of its associated deubiquitinases alters TCR signaling, resulting in increased GR expression, which could be rescued by TCR activation. We also demonstrate that modulation of LCK activity decreases IL-7R expression (Fig. 4E and tables S4 and S5), which is consistent with the finding that highlights inhibition of IL-7R in GC-resistant T-ALL (*65*), demonstrating that these signaling pathways are intertwined. Further studies regarding identification of a potential effector, downstream of TCR signaling, which might transcriptionally regulate GR expression, are warranted. In addition, how changes in LCK levels, modifications, or conformation affect the activity or localization of GR also warrants further investigation.

Furthermore, our findings that increased connectivity between promoter and IGR region of GR targets upon USP7 inhibition in the context of GCs highlight the critical role of chromatin connectivity on GR targets activation, which also determines the sensitivity and response to the GCs (*51*). On the basis of these results and given the wide use of GCs in autoimmune diseases and their various side effects, it might be interesting to study the effect of GCs in combination with LCK inhibition in this context (*66*, *67*).

Oncogenic functions of USP11 have been studied in cancer, both in solid tumors by controlling of DNA damage response, regulation of tumor suppressors, and apoptosis (*36*, *68*–*70*) and in blood tumors via the control of protein synthesis (*34*). A clinical trial in relapsed T-ALL showed that mitoxantrone, a type II topoisomerase inhibitor that also inhibits USP11, increased progression-free and overall survival compared with idarubicin (*71*, *72*). *Usp11* knockout animals are viable, suggesting that USP11 is not required for organismal homeostasis and that targeting USP11 might be a therapeutic modality in T cell leukemia. In addition, previous studies showed that USP7 inhibition can be a valid therapeutic modality in T-ALL (*22*, *73*). Of note, USP7 mutations that are potentially loss of function have been reported in TAL1-positive cases of T-ALL (*74*, *75*). Although the contribution of these mutations to pathways such as GR, IL-7, and NOTCH1 warrants investigation, a synergistic effect of USP7 haploinsufficiency with aberrant TAL-1 activation on T-ALL was observed, suggesting that the levels of USP7 are critical in TAL-1-positive cases (*23*). One should silence or inhibit USP7 substantially to block leukemia due to a potential happloinsufficient tumor suppressor role of USP7 in some disease contexts. In this study, we demonstrate that modulation of LCK activity via LCK or USP7 inhibition or USP11 silencing renders cells more sensitive to DXM and cooperates with GCs to block T-ALL via the induction of apoptosis.

## METHODS

### Patient samples, mice, and cells

All mice were housed in a barrier facility, and procedures were performed as approved by the Northwestern University Institutional Animal Care and Use Committee (protocol Ntziachristos no. IS00002058 and Mazar no. IS00000556) and the animal welfare committee of Ghent University. The cells are periodically tested for the presence of mycoplasma using the Lonza Walkersville MycoAlert Mycoplasma Detection Kit. The cell lines have been authenticated using short-tandem repeats profiling (JURKAT and DND41) or using polymerase chain reaction (PCR) to detect the TCRb-NOTCH1 translocation (*TCRBJ2S4CUTLL1F:5*′-GGACCCGGCTCTCAGTGCT-3′, *NOTCH1CUTTL1R:*5′-​TCCCGCCCTCCAAAATAAGG-3′). Human “thymocytes” refer to magnetically isolated thymocytes from human thymi derived from pediatric heart surgery (age from 7 days to 6 months). This refers to the previously published study by our groups; see the “Sorting of human thymocyte populations from human thymus” section in (*76*). Human CD3^+^, CD8^+^, and CD4^+^ T cells were purchased from AllCells.com (Alameda, CA) or from Astarte Biologics (Bothell, WA). Primary human T-ALL samples were collected under an informed consent after the study, and potential consequences were explained to the patients under the supervision of the Institutional Review Board of Padova University, the Associazione Italiana Ematologia Oncologia Pediatrica, the Berlin-Frankfurt-Münster (AIEOP-BFM) ALL 2000/2006 clinical trials, and the Institutional Review Board of Ghent University. The risk group is classified by detection of minimal residual disease (MRD) based on detection of leukemic blasts with patients. Patients were considered MRD standard risk if MRD was negative at days 33 and 78, medium risk if MRD was positive either at day 33 or 78 with up to 1 leukemic cell per 1000 healthy cells (< 10^−3^) on day 78, and high risk if more than 1 leukemic cell per 1000 healthy cells (>10^−3^) is detected on day 78. The study was registered at http://clinicaltrials.gov (“Combination chemotherapy based on risk of relapse in treating young patients with acute lymphoblastic leukemia,” protocol identification nos. NCT00430118 and NCT00613457). Mouse thymocytes were isolated from young animals (4 to 6 weeks old) and represent total population of thymocytic T cells (mainly including double negative and double positive for CD4 and CD8 markers). The *Usp11* knockout mice were purchased from European Mouse Mutant Archive-Infrafrontier and the Wellcome Trust Sanger Institute (em: 08053; http://informatics.jax.org/allele/MGI:4419682). The insertion of the L1L2_Bact_P cassette created a deletion of size 2743 starting at position 20578261 and ending at position 20581004 of chromosome X at *Usp11* allele (Genome Build GRCm39). *Mx1-Cre Usp7* conditional knockout mice (*50*) were gifts from D. Fang’s and W. Gu’s laboratories. Table S6 contains patient samples used for ex vivo and in vivo treatments. Information of patients nos. 20 to 26 was previously described (*77*). The survival cohort analyzed contains 264 T-ALL patients from St. Jude (the pediatric cancer genome project, PeCan) stratified according to T-ALL subgroup, maturation stage, and whether they belong to the early T cell progenitor status (see https://ocg.cancer.gov/programs/target/data-matrix). For the DXM-treated *Usp7* knockout model in vivo study (Fig. 5H), on the basis of our in vitro data, we determined that seven animals per group provide 80% power to detect differences of 1.3 SDs in circulating lymphoblasts between the four groups at a significance level of α = 0.05. After transplantation, the animals were treated with polyinosinic/polycytidylic acid (poly:I/C), coupled to randomization in each of the wild-type and conditional knockout groups, and with DXM.

### Antibodies and reagents

The following antibodies were used for Western blotting, immunofluorescence, or immunoprecipitation: mouse anti-actin (Millipore, clone C4), rabbit anti-USP11 (Abcam, ab109232), mouse anti-USP11 (Santa Cruz Biotechnology, sc-365528), rabbit anti-USP11 (Proteintech, 22340-1-AP), rabbit anti-LCK [Cell Signaling Technology (CST), 2752], mouse anti-LCK (Santa Cruz Biotechnology, sc-433), mouse anti-GR (Santa Cruz Biotechnology, sc-393232), rabbit anti-ZAP70 (CST, 2705), rabbit anti-ZAP70 (Tyr^319^) (CST, 2701), rabbit anti–SHP-1 (Bethyl Laboratories, A304-969A), rabbit anti–SHP-1 (Tyr^564^) (CST, 8849), rabbit anti-BIM (CST, 2890), rabbit anti-CYLD (CST, 8462), rabbit anti–phospho-Lck (Tyr^505^) (CST, 2751), rabbit anti-USP7 (Bethyl Laboratories, A300-033A-7), rabbit anti-cleaved NOTCH1 (Val^1744^) (CST, 4147), rabbit anti–phospho-Src family (Tyr^416^) (CST, 2101), rabbit anti–glyceraldehyde-3-phosphate dehydrogenase (GAPDH; CST, D16H11), rabbit anti-histone H3 (Abcam, ab12579), rabbit anti-HSP90 (Santa Cruz Biotechnology, sc-69703), hemagglutinin antibody (C29F4, cat. no. 3724), and FLAG antibody (Sigma-Aldrich, F1804). All antibodies for flow cytometry were from eBioscience. Secondary antibodies for immunofluorescence were goat anti-rabbit immunoglobulin G (IgG) H&L (Alexa Fluor 488) (Abcam, ab150077) and goat anti-mouse IgG H&L (Alexa Fluor 647) (Thermo Fisher Scientific, A-21235). Secondary antibodies for Western blots were horseradish peroxidase–conjugated anti-rabbit and anti-mouse IgG (GE Healthcare). DXM was purchased from Sigma-Aldrich. Chlorhexidine and doxycycline reagent were purchased from Thermo Fisher Scientific. Dynabeads human T-Activator CD3/CD28 and cell fractionation kit were purchased from Thermo Fisher Scientific. The latest generation of Progenra inhibitors (USP7i), P217564, was a gift from Progenra (Malvern, PA). USP7 inhibitor (P5091) generated by Progenra was also used yielding similar results to P217564. Dasatinib, bosutinib, and WH-4-023 were purchased from Selleckchem (Houston, TX). USP11 peptide ligand was a gift from I. Dreveny’s laboratory. Hi-C kits were purchased from Arima Genomics.

### TMT-labeled total proteome and phosphoproteome

Sample preparation for quantitative MS was performed as previously described (*78*). For the isobaric labeling for total proteome, 100 μg of peptide from each sample was labeled with tandem mass tag (TMT11) reagents (1:4; peptide:TMT label) (Thermo Fisher Scientific) for 2 hours at 25°C. Modification of tyrosine residues with TMT was reversed by the addition of 5% hydroxyl amine for 15 min at 25°C. The reaction was quenched with 0.5% trifluoroacetic acid (TFA), and a small, equal aliquot of each sample was combined to check for TMT labeling efficiency and total signal to noise per channel. Final samples were combined to equalize total TMT signal to noise across all channels, desalted on a C18 solid-phase extraction cartridge, and dried by SpeedVac. TMT-labeled peptides were solubilized in 5% acetonitrile (ACN)/10 mM ammonium bicarbonate (pH 8.0) and separated on an Agilent 300 Extend C18 column (3.5-μm particles, 4.6-mm inside diameter and 250 mm in length) with an Agilent 1260 binary pump coupled to a photodiode array detector (Thermo Fisher Scientific). A 45-min linear gradient from 10 to 40% ACN in 10 mM ammonium bicarbonate (pH 8.0, flow rate of 0.6 ml/min) separated the peptide mixtures into a total of 96 fractions (36 s), which were consolidated into 24 final fractions in a checkerboard fashion and dried by SpeedVac. Every other fraction was desalted via Stage Tip and redissolved in 5% formic acid (FA)/5% ACN for liquid chromatography tandem MS (LC-MS/MS) analysis.

For the enrichment and isobaric labeling of phosphopeptides, 800 μg of each sample was enriched for phosphopeptides using the High-Select Fe-NTA phosphopeptide enrichment kit (Pierce) according to the manufacturer’s instructions. Eluted phosphopeptides were dried by SpeedVac, resuspended in 0.1% TFA, and desalted on 10 mg of SOLA HPR solid-phase extraction cartridges (Thermo Fisher Scientific). Peptides were dried by SpeedVac, labeled with TMT11 reagents, and multiplexed as described above. Phospho-tyrosine–containing peptides were enriched from the desalted sample using the P-Tyr-1000 rabbit monoclonal antibody (CST) according to the manufacturer’s instructions. Eluted peptides were cleaned by stage tip into an MS sample vial and dried by SpeedVac. The nonbound fraction was retained, desalted by SepPak, dried by SpeedVac, and fractionated by basic reversed-phase high-performance LC, as described above, into a 96-well plate. Each column was combined for a total of 12 final fractions, which were dried by SpeedVac, cleaned by stage tip, and resuspended in 5%FA/5% ACN for MS analysis.

### Liquid chromatography–mass spectrometry

TMT11 multiplexed protein samples were analyzed with an LC-MS3 data collection strategy on an Orbitrap Fusion mass spectrometer (Thermo Fisher Scientific) equipped with a Proxeon Easy nLC 1000 for online sample handling and peptide separations as previously described (*79*, *80*).

### Real-time primers

The real-time primers are follows: *USP11*-human: (forward) 5′-CGTTTCCGGGACCAGAATCC-3′ and (reverse) 5′-CATCGCCGTCCGTTCTCTTC-3′; *NR3C1*-human: (forward) 5′-GGAATAGGTGCCAAGGATCTGG-3′ and (reverse) 5′-GCTTACATCTGGTCTCATGCTGG-3′; *ACTB*-human: (forward) 5′-CTCGCCTTTGCCGATCC-3′ and (reverse) 5′-GGGGTACTTCAGGGTGAGGA-3′; *BCL2L11*-human: (forward) 5′-GGTCCTCCAGTGGGTATTTCTCTT-3′ and (reverse) 5′-ACTGAGATAGTGGTTGAAGGCCTGG-3′.

### Cell transfection and virus production

Transfection and virus production were performed as previously described (*81*). The following shRNAs (Sigma-Aldrich, MISSION system) were used: *shLCK.1*: 5′-CCGGGAATGGGAGTCTAGTGGATTTCTCGAGAAATCCACTAGACTCCCATTCTTTTTTG-3′ (TRCN0000426292, NM_005356.3-1098s21c1); *shLCK.2*: 5′-CCGGGCATGAACTGGTCCGCCATTACTCGAGTAATGGCGGACCAGTTCATGCTTTTTG-3′ (TRCN0000335893, NM_005356.3-744s21c1); *shUSP11.1*: 5′-CCGGCCGTGACTACAACAACTCCTACTCGAGTAGGAGTTGTTGTAGTCACGGTTTTT-3′ (TRCN0000011090, NM_004651.2-1800s1c1); *shUSP11.2*: 5′-CCGGCCGTGATGATATCTTCGTCTACTCGAGTAGACGAAGATATCATCACGGTTTTTG-3′ (TRCN0000315210, NM_004651.3-1695s21c1); *shUSP7*:5′-CCGGCCTGGATTTGTGGTTACGTTACTCGAGTAACGTAACCACAAATCCAGGTTTTT-3′; and nonmammalian shRNA control hairpin SHC002: 5′-CCGGCAACAAGATGAAGAGCACCAACTCGAGTTGGTGCTCTTCATCTTGT.

TGTTTTT-3′.

Inducible shRNAs from Horizon Discovery [*shUSP11* (SMARTvector Inducible Lentiviral)] were used: *shUSP11.a* V3SH11252-228470650 (clone ID: V3IHSMCG_8408300). TRIPZ inducible lentiviral non-silencing shRNAs (RHS4743) were used as a shRNA control.

### Intravenous xenograft and *Usp7* and *Usp11* knockout mice studies

All mice were housed in a barrier facility, and procedures were performed as approved by the Northwestern University Institutional Animal Care and Use Committee (protocols Ntziachristos no. IS00002058 and Mazar no. IS00000556). For CUTLL1 or JURKAT T-ALL intravenous studies, 1 million cells in 100 μl of phosphate-buffered saline were injected into the tail vein of 8-week-old NOD.Cg-Prkdcscid male mice (no. 005557, Jackson Laboratories, Portage, MI). Animals were monitored by in vivo imaging system (IVIS) every 3 days for luciferase signal detection. IVIS images were taken using the IVIS Spectrum in vivo imaging system (PerkinElmer). These experiments were performed by Center for Developmental Therapeutics of Northwestern University.

For complete blood counts analysis, blood (50 μl) was collected from the tail vein in EDTA-coated tubes and analyzed by a Hemavet 850 complete blood counter (Drew Scientific). Thymocyte isolation from mice and flow cytometry analysis were performed as previously described (*81*). The NOTCH1-induced mouse T-ALL model was generated as previously described (*81*).

For the USP11 mutant plasmid, the conserved 275 cysteine residue of the USP11 catalytic domain was replaced by a serine residue in the mutant USP11. For the USP7 mutant plasmid, the conserved 223 cysteine residue of the USP7 catalytic domain was replaced by a serine residue in the mutant USP7.

Cell growth and viability assays, apoptosis, drug synergism, cell cycle analysis, cell fractionation assay, ubiquitination assays, RNA isolation, RNA-seq, gel filtration, immunoprecipitation, immunoprecipitation–LC-MS/MS, ubiquitin Lys-ϵ-Gly-Gly (KεGG) assay, immunoblots, and RPPA were performed as previously described (*21*, *22*). Immunofluorescence was performed as previously described (*82*). Patient sample nos. 1 to 19 were cultured ex vivo as previously described (*28*), and patient sample nos. 20 to 26 were culture ex vivo as previously described (*77*). Hi-C was performed following the Arima Genomics kits.

### Chromatin immunoprecipitation–quantitative polymerase chain reaction

DND41 cells were treated with either dimethyl sulfoxide, 5 μM USP7i (p5091), 1 μM dexamethasone (DXM), or their combination for 6 hours. A total of 30 M cells (in 30 ml of medium; 1 M cells/ml) were cross-linked with 1% formaldhyde 10 min followed by a 5-min treatment with 0.125 M glycine. Cells were lysed, and the cross-linked DNA was sheared into 200– to 400–base pair fragments using the truChIP chromatin hearing kit (Covaris) and Covaris M220. One percent of the sheared DNA was used as input, and the remaining material was incubated with 5 μg of rabbit GR (G-5) antibody. The eluted DNA was used as input for quantitative PCR (qPCR) for promoter and IGR (primer sets 1 and 2) and negative control (non–GR-bound) loci (*83*).

### Bioinformatics analysis

RNA-seq reads were mapped to human genome hg19 using TopHat. Differential gene expression analysis was performed using the EdgeR package in R. Gene expression changes were visualized in heatmaps using the ggplot2 package in R. Enriched Kyoto Encyclopedia of Genes and Genomes pathways and gene ontology terms were identified using gene set enrichment analysis (*84*) or EnrichR (*85*). Bubble charts representing enrichment analysis were generated using the pathfindR package in R. Venn diagrams of overlaps were generated using an online Venn diagram generator (https://meta-chart.com/venn​). The raw data for the cohort of pediatric T-ALL samples showing an increased USP11 expression and the KGG study in regard to USP7 inhibition on T-ALL cells were analyzed from previous publication (*21*). Statistical quantification of Western blot results was analyzed by ImageJ. Synergy analysis was performed by using an online synergy generator (https://synergyfinder.fimm.fi). Hi-C analysis was performed as before (*59*). ATAC-seq was performed as before (*86*, *87*).

### Proteomics data analysis

Label-free quantification (LFQ), based on MS1 intensity or MS2 spectral count, is carried out by MaxQuant v1.6.0.16 (*88*, *89*). Proteins that were identified with two peptides (plus localization probability of 75% for phosphopeptides) are further investigated for relative quantification. MaxQuant LFQ intensities are converted to log_2_ values using Perseus software (version 1.6.13.0) (*90*), and differences in relative expression of proteins by fold change or *t* test were used to test statistical significance with differences considered as statistically significant for *P* values of <0.05 between test groups. When applicable, volcano plots for *t* test significance were obtained by plotting the values.

TMT reporter ion quantification and validation of identified peptides and proteins were performed by Scaffold software (version 5.0, Proteome Software Inc., Portland, OR). Intensity of reporter ions was calculated by Scaffold Q+. A 1% false discovery rate (FDR) cutoff was applied at the peptide level. Only proteins with a minimum of two peptides above the cutoff were considered for further study. Analysis of variance test with Benjamini and Hochberg FDR correction is applied to the comparison among the conditions using a 0.05 threshold for statistical significance.

### Analysis of data from publicly available databases

Analysis of microarray data from Gene Expression Omnibus (GEO) was done using the National Center for Biotechnology Information GEO2R online tool for microarray analysis. Quantile normalization was used to process microarray data. Adjusted *P* value calculations were done using the Benjamini-Hochberg option. A *P* value of <0.05 was statistically significant. Gene essentiality data for cancer cell lines were obtained from the Project Achilles CRISPR-Cas9 screening dataset (https://depmap.org/portal/download/; 2019 Quarter 2 release). Essentiality of individual genes is represented as the inverse of the CERES score for that gene (*91*). Visualization of gene essentiality data was achieved in Python (version 3.6.4, Anaconda Inc.)​ using the modules Pandas (v0.23.4) and Seaborn (v0.9.0). Survival curves were generated using GraphPad. Kaplan-Meier analysis and Cox proportional hazard models were used for survival analysis by using R package “survminer” (v0.4.6). Missing data were coded and excluded from the analysis. *P* < 0.05 was considered to indicate a statistically significant difference.

## Notes

View/request a protocol for this paper from *Bio-protocol*.
