# Supplementary material for: Oncogenic deubiquitination controls tyrosine kinase signaling and therapy response in acute lymphoblastic leukemia
Source: Sci Adv. 2022 Dec 9;8(49):eabq8437. doi: 10.1126/sciadv.abq8437 (PMC9733937; doi:10.1126/sciadv.abq8437)
Supplement: Supplementary file 1 — Figs. S1 to S16 [file sciadv.abq8437_sm.pdf]

Science Advances
AAAS

Supplementary Materials for

Oncogenic deubiquitination controls tyrosine kinase signaling and therapy response in acute lymphoblastic leukemia

Qi Jin *et al.*

Corresponding author: Panagiotis Ntziachristos, pntziachr@gmail.com

Sci. Adv. **8**, eabq8437 (2022)
DOI: 10.1126/sciadv.abq8437

The PDF file includes:

Figs. S1 to S16
Legends for tables S1 to S6

Other Supplementary Material for this manuscript includes the following:

Tables S1 to S6

Supplementary Figure 1. Jin, Gutierrez, et al., 2022

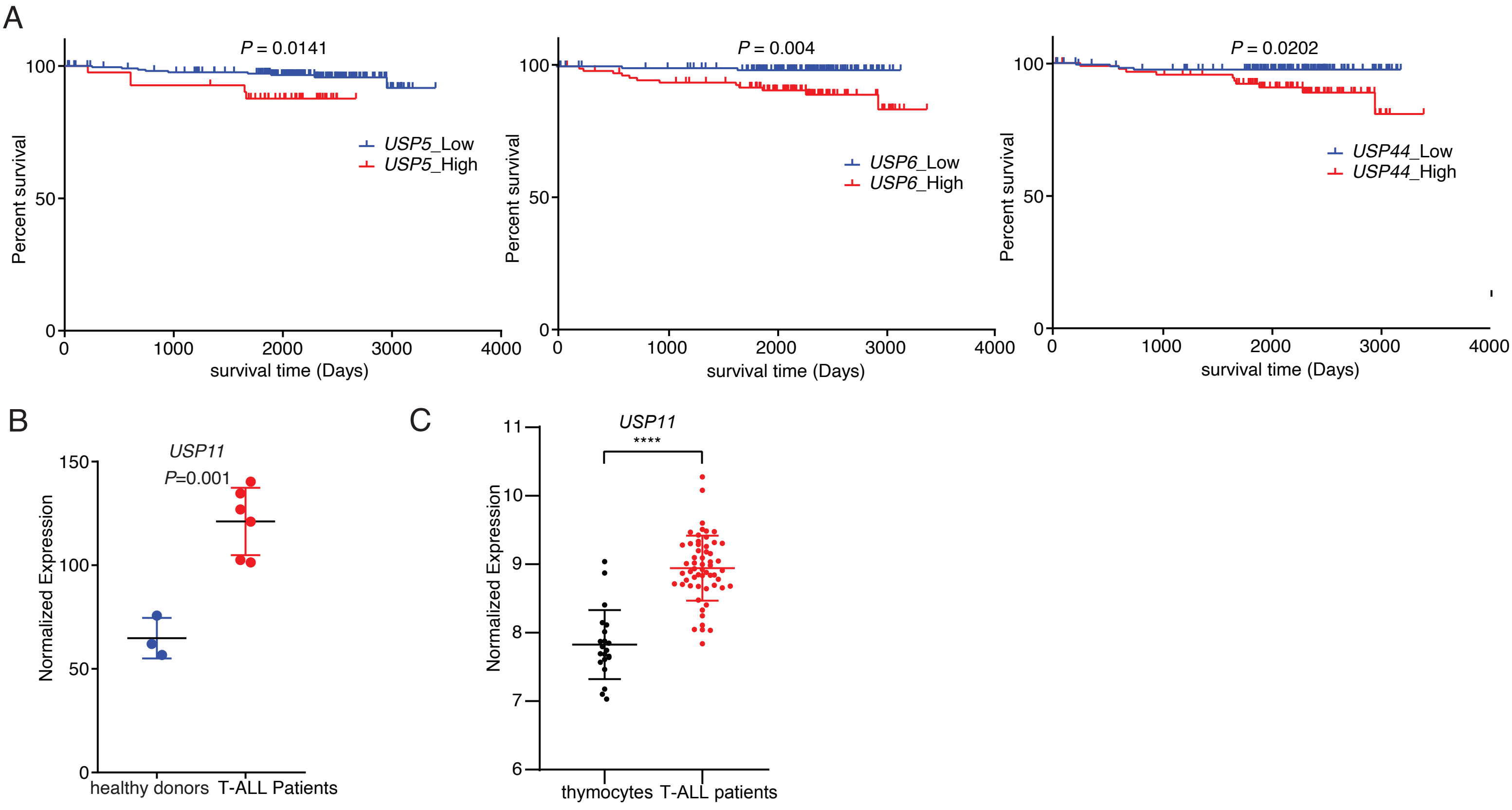

Supplementary Figure 1. Analysis of USP levels in cancer. **A**, Survival analysis by gene expression for USP5 (left panel), USP6 (middle), and USP44 (right panel) in T-ALL patients based on PeCan data. **B**, Graph showing the expression of USP11 in T-ALL patient samples (*n*= 6 samples) and control $CD3^+$ T cells from healthy donors (*n*= 3 samples). **C**, Graph showing the expression of USP11 in T-ALL samples (*n* = 57 samples) and physiological thymocyte subsets (*n* = 21 samples; 7 T cell subsets derived from 3 donors in total). ****P<0.0001.

Supplementary Figure 2. Jin, Gutierrez, et al., 2022

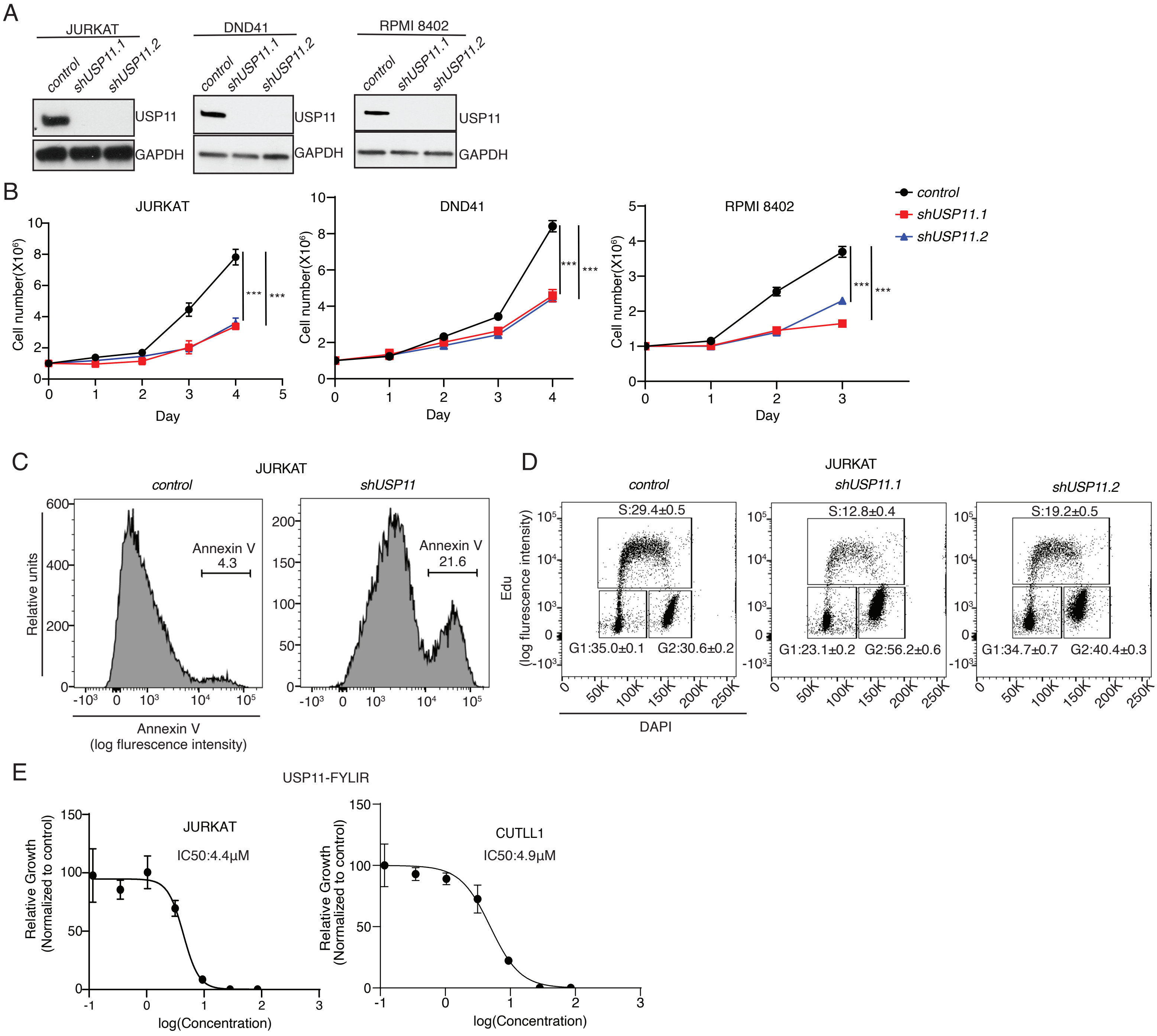

Supplementary Figure 2. Targeting *USP11* inhibits T-cell leukemia growth *in vitro*. A, Immunoblot detection of USP11 protein levels in control- and *shUSP11*-expressing JURKAT, DND41, and RPMI 8402 cells. GAPDH was used as the loading control. **B**, Growth curves of control- and *shUSP11*-expressing JURKAT, DND41, and RPMI 8402 cells over a period of 4 days (n=3, *** P<0.001). **C**, Annexin V staining (72 h) of JURKAT cells that express either control shRNA or *shUSP11*. A representative example of 3 experiments is shown. **D**, Edu staining (72 h) of JURKAT cells that express either control shRNA or *shUSP11*. The mean ±s.d. from two representative experiments is shown. **E**, IC_{50} curve of USP11–FYLIR (the USP11 peptide ligand) in JURKAT (left panel) and CUTLL1 (right panel) cells.

Supplementary Figure 3. Jin, Gutierrez, et al., 2022

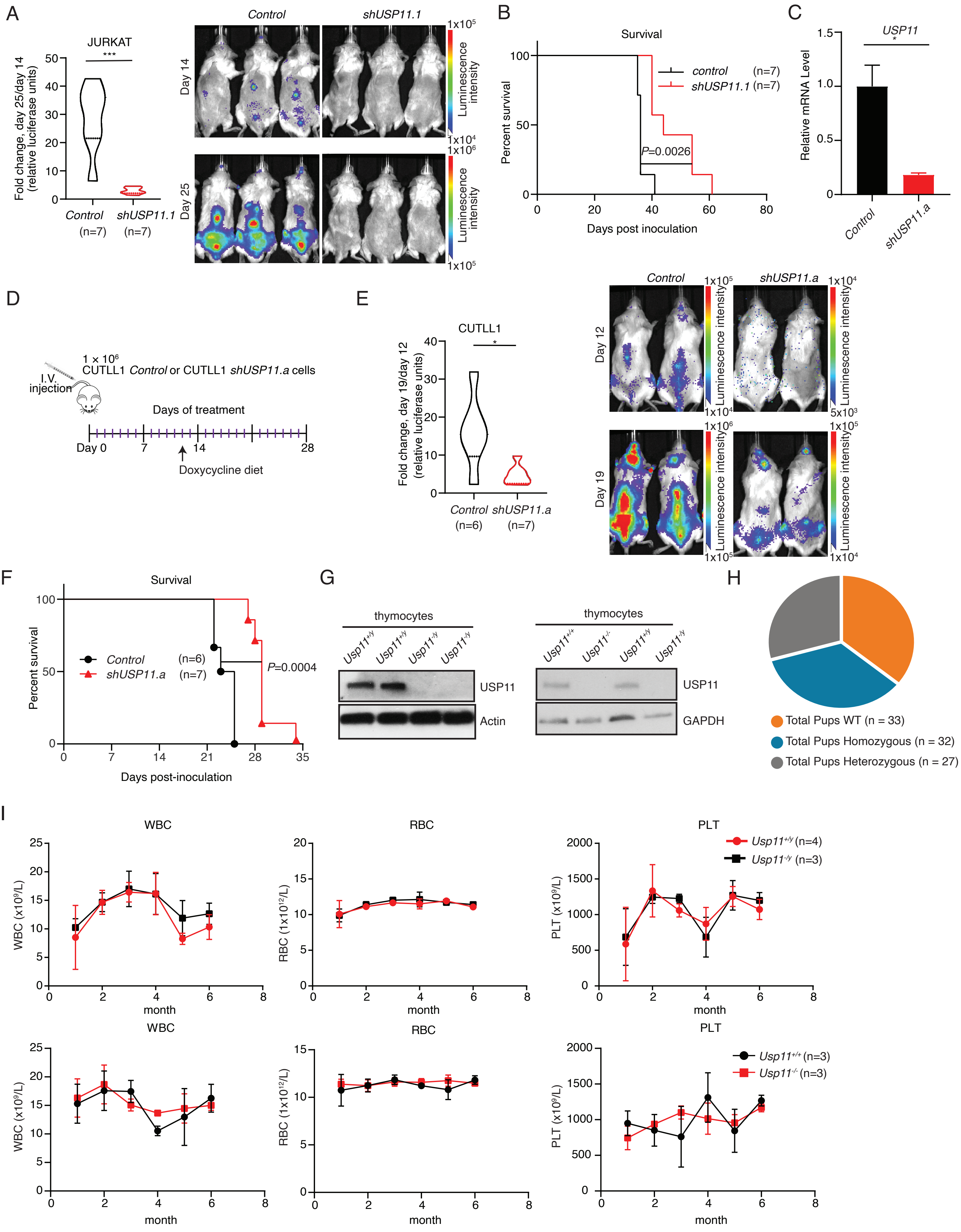

Supplementary Figure 3. Targeting *USP11* impedes T-cell leukemia growth while sparing normal hematopoiesis *in vivo.* **A**, Luciferase-expressing JURKAT cells were transduced with lentiviral vector expressing a control hairpin RNA or *shUSP11.1*, selected using puromycin for a period of 3 days, and injected intravenously into immunocompromised mice. Leukemic burden was assessed twice per week by bioluminescence measurements. Relative bioluminescence intensity is shown for three representative mice per group on days 14 and 25 of treatment (right panel). The fold change in total flux from days 14 and 25 is shown on the left (*** P<0.001). **B**, Survival analysis of mice transplanted with control hairpin RNA or *shUSP11.1*-expressing JURKAT cells. P value was calculated by Log-rank (Mantel-Cox) test. **C**, RT-PCR of USP11 in luciferase-expressing CUTLL1 cells transduced with a doxycycline-inducible control shRNA and *shUSP11.a in vitro.* P value was calculated by t-test (*control*, n=2; *shUSP11.a*, n=2, * P<0.05). **D**, Luciferase-expressing CUTLL1 control shRNA and *shUSP11.a* were injected into immunocompromised animals and, upon detection of luminescence, doxycycline was administered to the animals. **E**, Fold change in total flux from days 12 and 19 are shown (* P<0.05) (left panel). Relative bioluminescence intensity is shown for two representative mice per group on days 12 and 19 of treatment (right panel). **F**, Survival analysis of mice transplanted with control hairpin RNA or *shUSP11.a*-expressing CUTLL1 cells. P value was calculated by Log-rank (Mantel-Cox) test. **G,** Representative western blot of USP11 in the indicated mouse genotypes. **H,** Viability Primary Screen phenotypic assay was performed on a mutant strain carrying the *Usp11*$^{\mathrm{tm1(KOMP)Wtsi}}$ allele. Data was accessed on the website https://www.mousephenotype.org/data/charts?accession=MGI:2384312&parameter_stable_id=IMPC_VIA_001_001&parameter_stable_id=IMPC_VIA_063_001&parameter_stable_id=IMPC_VIA_064_001&parameter_stable_id=IMPC_VIA_065_001&parameter_stable_id=IMPC_VIA_066_001&parameter_stable_id=IMPC_VIA_067_001. The charts above show the proportion of wild type, heterozygous, and homozygous offspring. **I**, Peripheral blood analysis of *Usp11*$^{+/y}$, *Usp11*$^{-/y}$, *Usp11*$^{+/+}$, and *Usp11*$^{-/-}$ mice that were monitored for a period of 6 months. White blood cells (WBC, left panel), red blood cells (RBC, middle) and platelet counts (PLT, right panel) were analyzed monthly.

Supplementary Figure 4. Jin, Gutierrez, et al., 2022

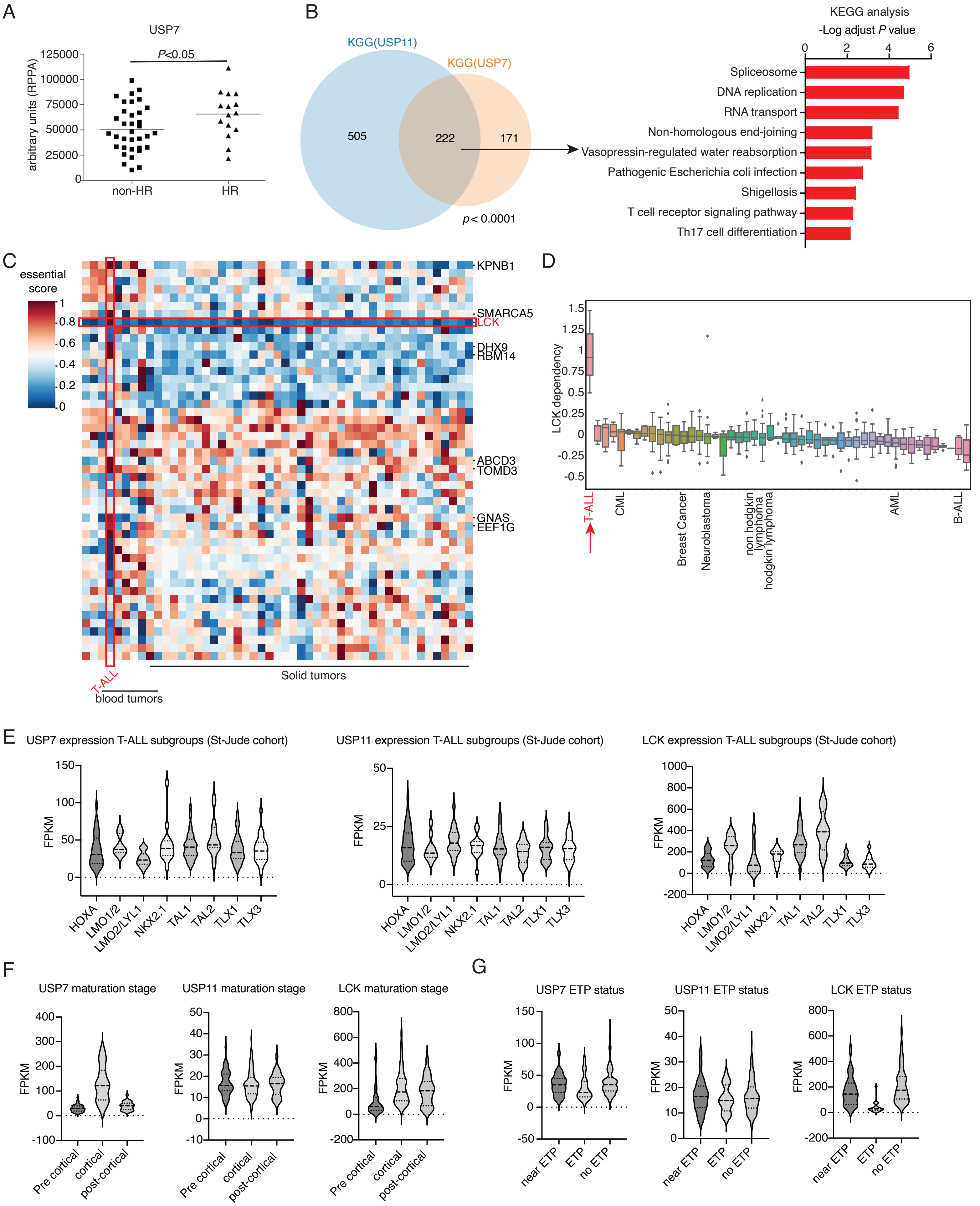

Supplementary Figure 4. Identification of LCK as a critical substrate of USP11 and USP7. **A,** RPPA analysis of USP7 in non-high-risk and high-risk groups of T-ALL patients (Mann-Whitney t test, *P*=0.01). The risk group is classified by MRD. **B**, Analysis of the KGG MS data for USP11 and USP7 (left panel) and KEGG analysis for overlapping proteins (right panel). **C,** Heatmap showing relative essentiality of the overlapping genes (*n*=51) from Figure 3B. Essentiality data, reflecting the importance of individual genes for cellular fitness, was obtained from the Project Achilles CRISPR/Cas9 screening dataset of 563 cancer cell lines. **D,** Essentiality score for LCK amongst different cancer types from Project Achilles. A gene essentiality score of 1 is typical for genes considered pan-essential. T-ALL and other representative cancer types are shown. **E-G,** Violin plots representing expression levels of USP7, USP11 and LCK that were retrieved from RNA sequencing data of 264 T-ALL patients. T-ALL patients were subdivided according to T-ALL subgroups (**E)**, maturation stage (**F**) and ETP status (**G)**. Except for USP7 being more prevalent in cortical T-ALLs and LCK being lowly expressed in ETP and more immature T-ALLs, all USP7/USP11/LCK complex members are being expressed in all T-ALL subgroups and developmental stages.

Supplementary Figure 5. Jin, Gutierrez, et al., 2022

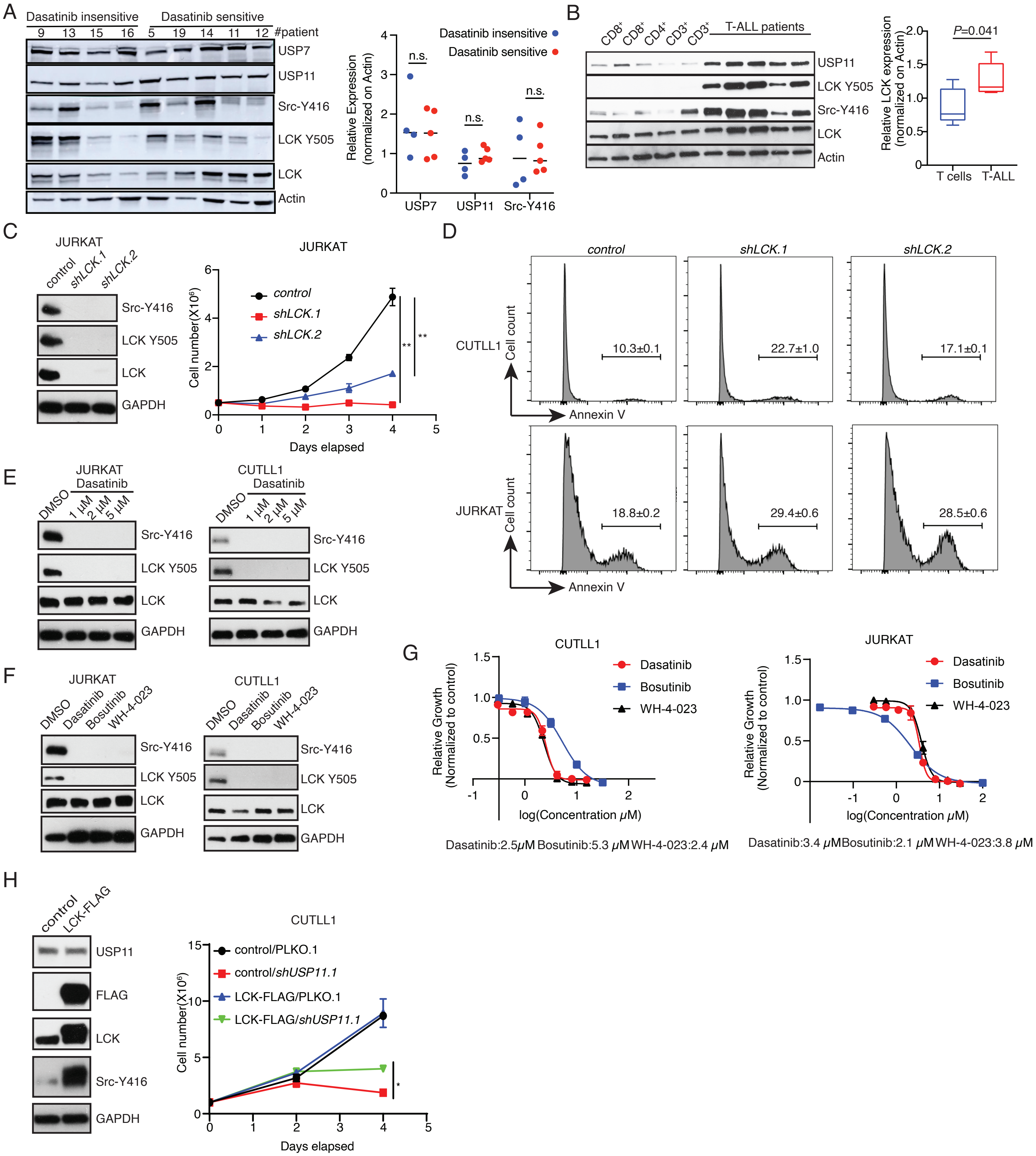

Supplementary Figure 5. LCK and its activity are essential for T-cell leukemia maintenance. **A,** Immunoblot detection of USP7, USP11, LCK, LCK phospho-Y505, Src phospho-Y416, and GAPDH (left panel) in dasatinib sensitive or dasatinib insensitive T-ALL patient samples described in Fig. 3D. Statistic quantification analysis were shown in right panel. **B,** Immunoblot detection of USP11, LCK, LCK phospho-Y505, Src phospho-Y416, and actin in T-ALL patients (n=5) and normal $CD8^+$ (n=2), $CD3^+$ (n=2), and $CD4^+$ T cells (left panel). Quantification of LCK bands (right panel) is shown. **C,** Immunoblot detection of LCK, LCK phospho-Y505, Src phospho-Y416, and GAPDH (left panel) as well as growth curves of *shLCK*-expressing JURKAT cells over a period of 4 days (*n*=3, right panel, ***P*<0.01). **D,** Annexin V staining of CUTLL1 (top panel) and JURKAT cells (bottom panel) that expressed either a control hairpin RNA, *shLCK.1* or *shLCK.2*. **E**, Immunoblot detection of LCK, LCK phospho-Y505, Src phospho-Y416, and GAPDH (upon treatment as indicated in the figure) for 24 h in JURKAT (left panel) and CUTLL1 cells (right panel). **F,** Immunoblot detection of LCK, LCK phospho-Y505, Src phospho-Y416, and GAPDH upon 24 h treatment of JURKAT (left panel) and CUTLL1 (right panel) cells with dasatinib, bosutinib, or WH-4-023. **G**, IC_{50} curve of dasatinib, bosutinib, and WH-4-023 in CUTLL1 (left panel) and JURKAT (right panel) cells. **H**, Immunoblot detection of FLAG, USP11, LCK, Src phospho-Y416, and GAPDH (left panel) of CUTLL1 cells that were transduced with control lentivirus vector and 3xFLAG-tagged LCK overexpressing vector (LCK-FLAG). Growth study over a period of 4 days of control and LCK-FLAG-overexpressing CUTLL1 cells transduced with control or *shUSP11.1* lentivirus (* *P*≤0.05).

Supplementary Figure 6. Jin, Gutierrez, et al., 2022

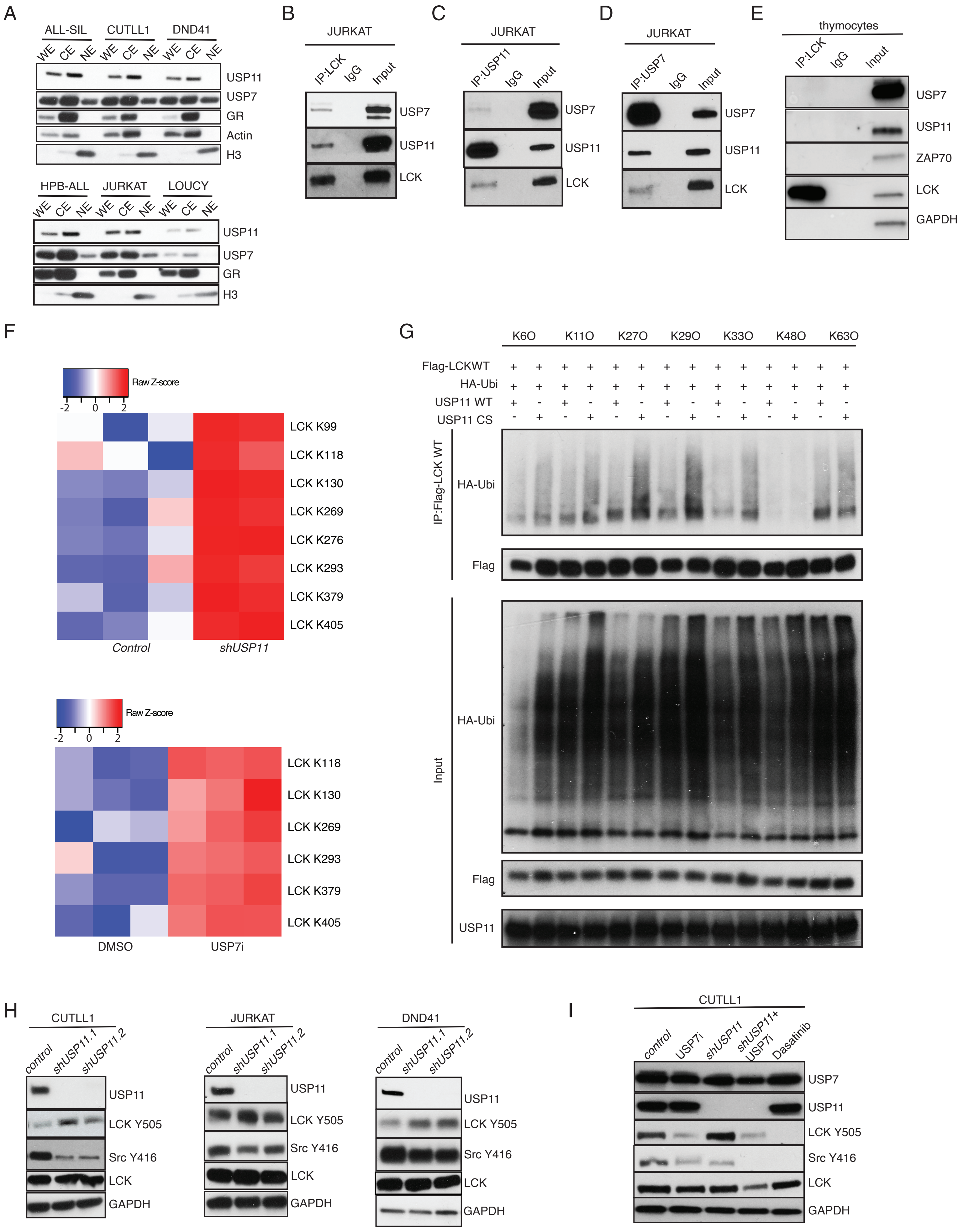

Supplementary Figure 6. LCK deubiquitination by USP11 and USP7 controls its activity. A, Representative immunoblots for of USP11, USP7, glucocorticoid receptor (GR), actin, and H3 in whole cell (WE), cytoplasmic (CE) and nuclear (NE) extracts of six T-ALL cell lines. **B-D**, Western blots following immunoprecipitation (IP) of LCK (**B**), USP11 (**C**), or USP7 (**D**) in JURKAT cells. USP7, USP11, and LCK were detected as indicated. **E**, Western blots following immunoprecipitation of LCK in mouse thymocytes. USP7, USP11, LCK, ZAP70 were detected as indicated. GAPDH was used as loading control. **F,** Heatmap showing differential lysine ubiquitination sites within LCK protein upon expression of *shUSP11* (top panel) and USP7i treatment (bottom panel, lysine ubiquitome studies). **G,** Effects of the indicated lysine-only ubiquitin mutants on USP11-mediated LCK deubiquitination. The specific K-only ubiquitin constructs presenting the wildtype lysines on positions ((K6O), K11O, K27O, K29O, K33O, K48O or K63O) with mutation on other lysines were utilized. 293T cells were transfected with the constructs as indicated, and LCK ubiquitination was analyzed. The ubiquitinated proteins were pulled down under denaturing conditions using FLAG beads. K6 indicates all 6 lysines combined. **H**, Immunoblot detection of USP11, LCK, LCK phospho-Y505, Src phospho-Y416, and GAPDH protein levels upon *shUSP11* expression in CUTLL1 (left panel), JURKAT (middle panel), and DND41 cells (right panel). **I**, Immunoblot detection of USP11, USP7, LCK, LCK phospho-Y505, Src phospho-Y416, and GAPDH protein levels in the indicated conditions.

Supplementary Figure 7. Jin, Gutierrez, et al., 2022

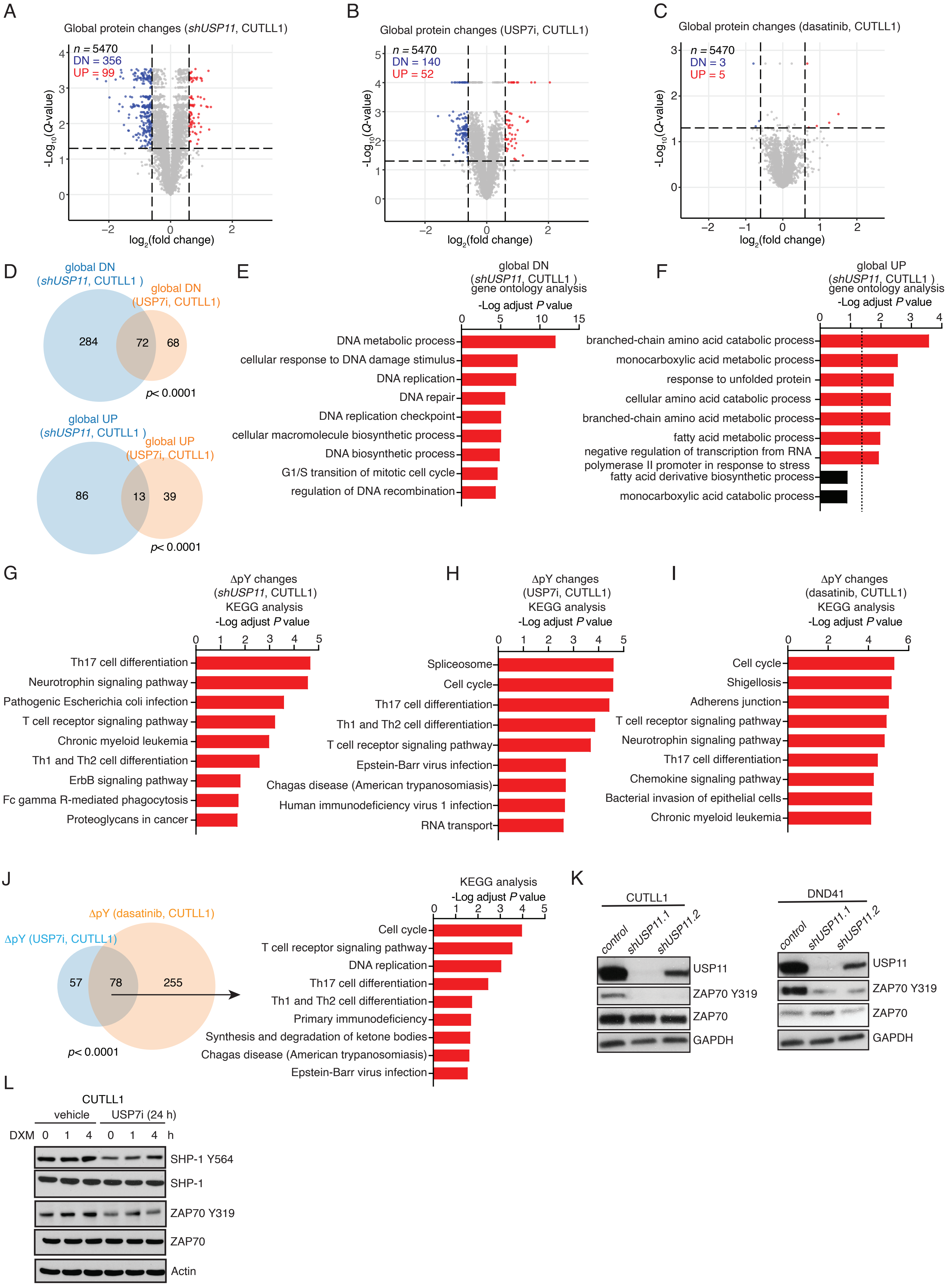

Supplementary Figure 7. Global protein and phospho-proteomics analysis upon *shUSP11* expression, dasatinib and USP7i treatment. A-C, Volcano plots showing proteomic changes in *shUSP11* vs. control*,* USP7i (5 μM) vs. control, and dasatinib (5 μM) vs. control in CUTLL1 cells. Multiple unpaired t-test (*P* value) followed by false discovery rate (FDR) (*Q* value) analysis (UP, increase; DN, decrease). **D**, Venn diagram showing overlap of increasing (top panel) or decreasing (bottom panel) global proteins in *shUSP11* vs. control and USP7i (5 μM) vs. control in CUTLL1 cells (UP, increase; DN, decrease). **E**, KEGG analysis of down-regulated global proteins in *shUSP11* vs. control group. **F**, KEGG analysis of up-regulated global proteins in *shUSP11* vs. control group. **G-I**, KEGG analysis of phospho-tyrosine containing peptide changes in *shUSP11* vs. control (**G**), USP7i (5 μM) vs. control (**H**), and dasatinib (5 μM) vs. control (**I**) in CUTLL1 cells. **J**, Venn diagram showing overlap of phospho-tyrosine containing peptide changes in USP7i vs. control and dasatinib vs. control in CUTLL1 cells (left panel). KEGG analysis of overlapping proteins (right panel). **K**, Immunoblot detection of USP11, ZAP70 phospho-Y319, ZAP70, and GAPDH levels in control or *shUSP11*- expressing CUTLL1 (left panel) or DND41 cells (right panel). **L**, Immunoblot detection of SHP-1 phospho-Y654, SHP-1, ZAP70 phospho-Y319, ZAP70, and actin upon dexamethasone (1 μM), USP7i (5 μM) or combination treatment in CUTLL1 cells at the indicated timepoints.

Supplementary Figure 8. Jin, Gutierrez, et al., 2022

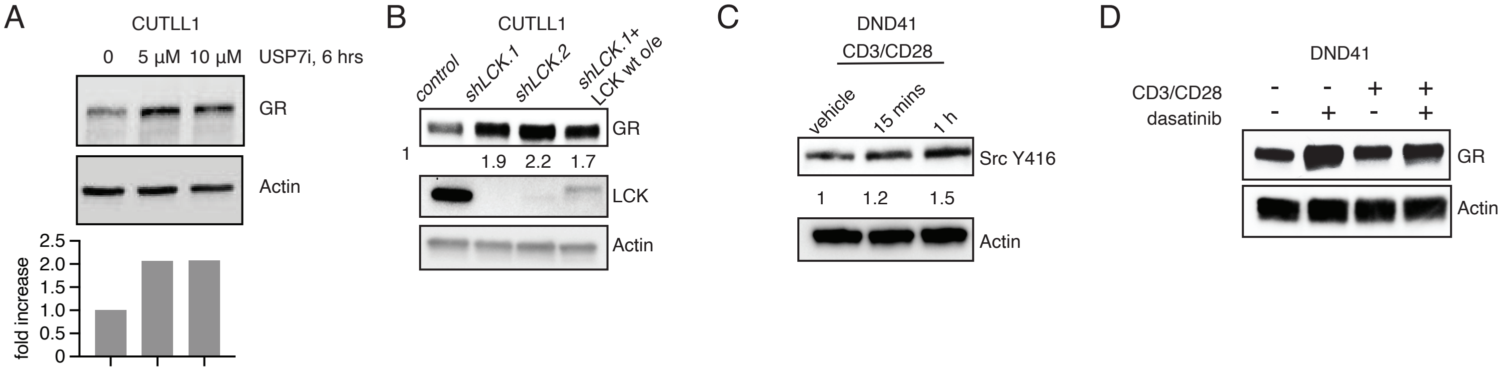

Supplementary Figure 8. LCK overexpression and TCR signaling activation partially rescue GR expression. A, Immunoblot detection of GR and actin levels in CUTLL cells after 5 μM or 10 μM USP7i treatment for 6 hours. **B**, Immunoblot detection of GR, LCK, and actin levels in CUTLL cells with expression of a control hairpin, *shLCK,* or *shLCK* in combination with overexpression (o/e) of wild-type (wt) LCK. **C**, Immunoblot detection of Src phospho-Y416 and actin upon treatment with CD3/CD28 in DND41 cells. Lower row of numbers indicates the relative density of the actin band vs. the Src band. **D**, Immunoblot detection of GR and actin upon 6 h treatment with CD3/CD28 beads, dasatanib (2 μM), or their combination in DND41 cells.

Supplementary Figure 9. Jin, Gutierrez, et al., 2022

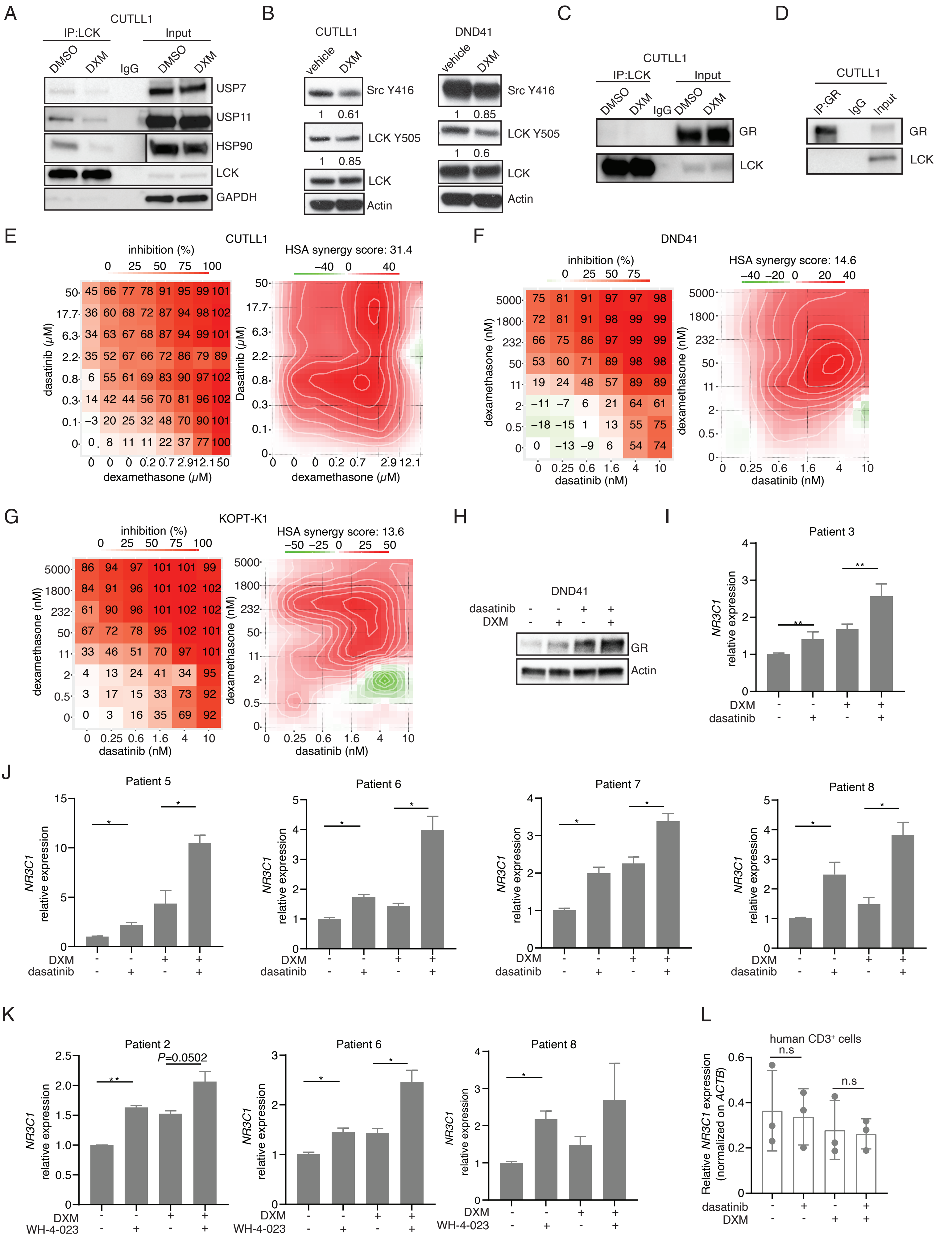

Supplementary Figure 9. LCK inhibition enhances the glucocorticoid response. A, Immunoblot detection following immunoprecipitation (IP) of LCK upon treatment with dexamethasone (DXM, 1 μM) for 24 h in CUTLL1 cells. USP7, USP11, HSP90, LCK, and GAPDH were detected. **B,** CUTLL1 (left panel) or DND41 (right panel) cells were treated with dexamethasone (DXM) as indicated followed by immunoblot detection of LCK, LCK phospho-Y505, Src phospho-Y416, and actin. Quantification values are shown below the corresponding bands. **C**, Immunoblot detection of GR and LCK following LCK immunoprecipitation (IP) with or without dexamethasone (1 μM, 24 h) treatment in CUTLL1 cells. **D**, Immunoblot detection of GR and LCK following GR immunoprecipitation in CUTLL1 cells. **E-G**, Synergy heatmaps for dasatinib and dexamethasone treatment for 72 h in CUTLL1 (**E**), DND41 (**F**) and KOPT-K1 (**G**) cells. HSA analysis is shown. **H**, Immunoblot detection of GR and actin upon treatment with dexamethasone (DXM, 100 nM), dasatinib (2 μM) or their combination for 6 h in DND41 cells. **I-J**, RT-qPCR analysis of *NR3C1* in T-ALL patient samples treated with DMSO, dexamethasone (100 nM), dasatinib (2 μM) or their combination for 6 h (* $P<0.05$). **K**, RT-qPCR analysis of *NR3C1* in T-ALL patient samples treated with DMSO, dexamethasone (100 nM), WH-4-023 (2 μM) or their combination for 6 h (* $P<0.05$, ** $P<0.01$). **L**, RT-qPCR analysis of *NR3C1* in human $CD3^+$ cells of three healthy donors, treated with dexamethasone (100 nM), dasatinib (2 μM) or their combination for 6 h.

Supplementary Figure 10. Jin, Gutierrez, et al., 2022

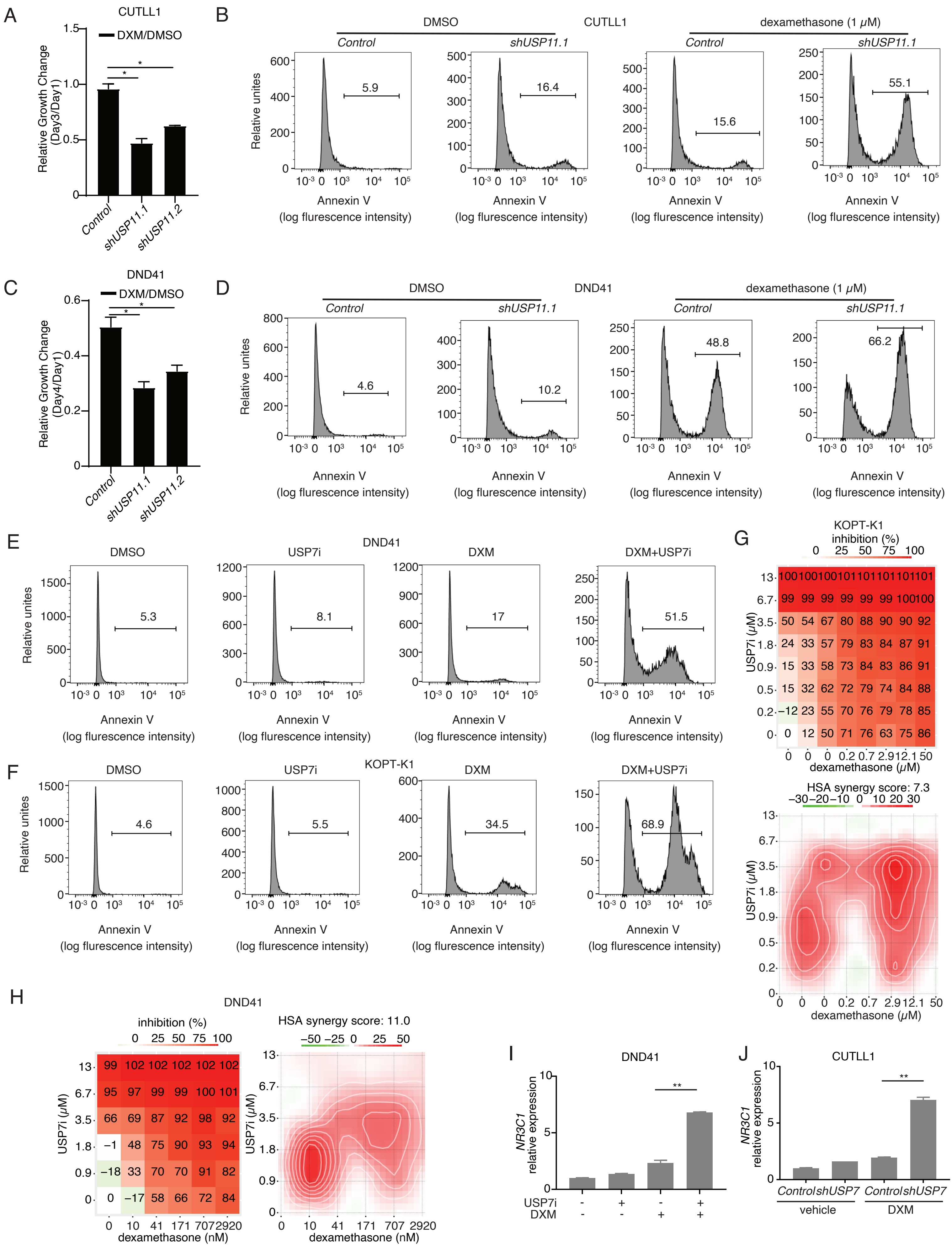

Supplementary Figure 10. USP7i or *shUSP11* sensitize the cells to glucocorticoids. A, CUTLL1 cells were transduced with control shRNA and *shUSP11*. Relative growth change upon dexamethasone (DXM, 1 μM) normalized to DMSO treatment is shown (n=3, * $P<0.05$). **B**, Annexin V staining (72 h) of CUTLL1 cells that expressed either control shRNA or *shUSP11.1*. Experiment was repeated three times and a representative example is shown. **C**, DND41 cells were transduced with control shRNA and *shUSP11*. Relative growth change upon dexamethasone (1 μM) normalized to DMSO treatment is shown (n=3, * $P<0.05$). **D**, Annexin V staining (72 h) of DND41 cells that expressed either control shRNA or *shUSP11.1*. Experiment was repeated three times and a representative example is shown. **E**, Annexin V (72h) staining of DND41 cells that were treated with USP7i (5 μM) or dexamethasone (1 μM). **F**, Annexin V (72h) staining of KOPT-K1 cells that were treated with USP7i (5 μM) or dexamethasone (1 μM). The experiment was repeated three times and a representative example is shown. **G**, Synergy heatmaps for the inhibition of cell growth of KOPT-K1 cells that were treated with USP7i and dexamethasone for 72 h. HSA analysis is shown. **H,** Synergy heatmaps for USP7 inhibitor and dexamethasone treatment for 72 hrs in DND41 cells. HSA analysis is shown. **I**, RT-qPCR analysis of *NR3C1* in DND41 cells treated with DMSO, dexamethasone (1 μM), USP7i (5 μM), or their combination (** $P<0.01$). **J**, RT-qPCR analysis of *NR3C1* in control and *shUSP7*-expressing CUTLL1 cells treated with vehicle or dexamethasone (1 μM) (** $P<0.01$).

Supplementary Figure 11. Jin, Gutierrez, et al., 2022

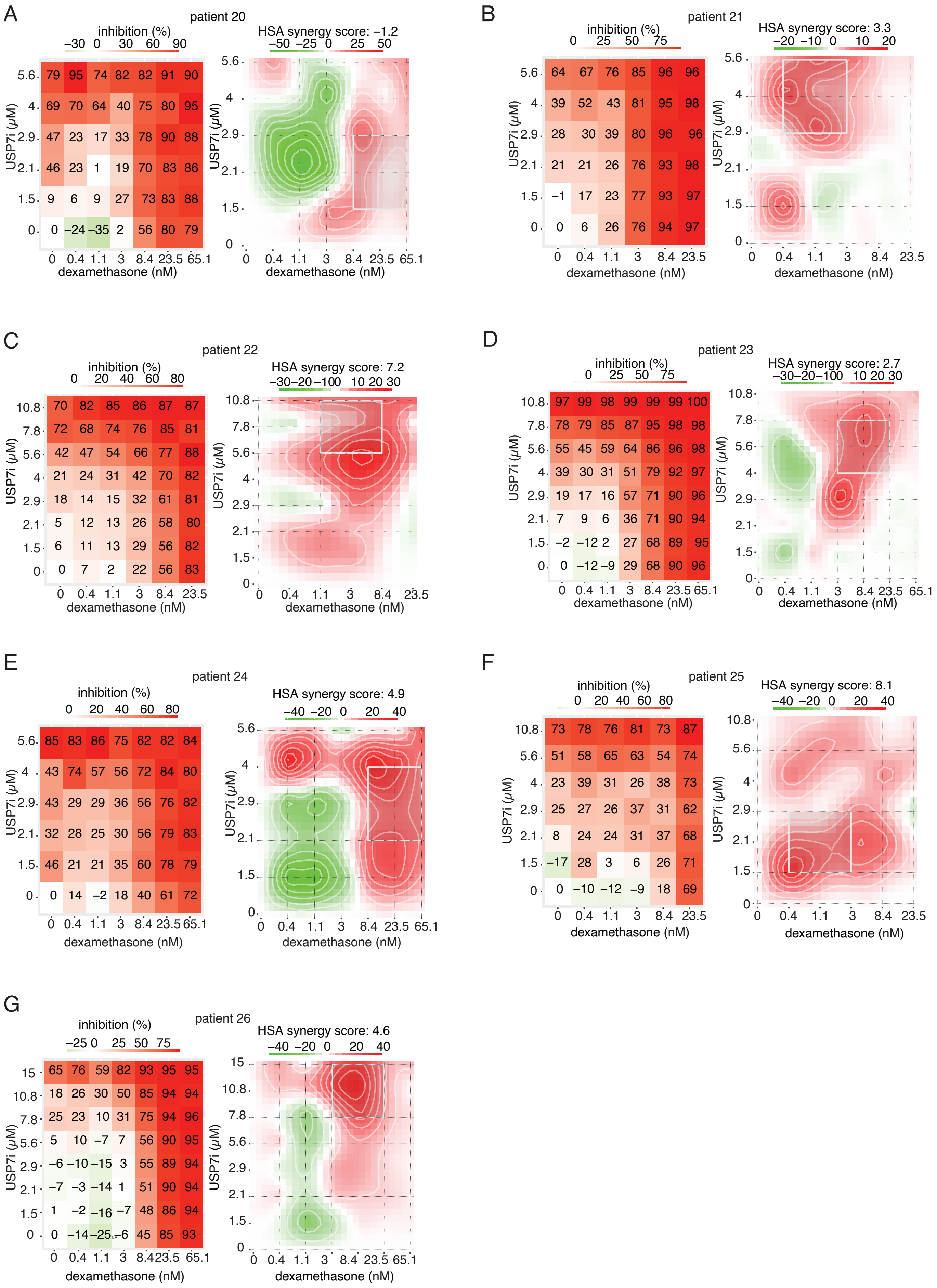

Supplementary Figure 11. Synergy between USP7i and dexamethasone in T-ALL patient samples. A-G, Synergy heatmaps for USP7 inhibitor and dexamethasone treatment for 5 days in 7 T-ALL patient samples. HSA analysis is shown.

Supplementary Figure 12. Jin, Gutierrez, et al., 2022

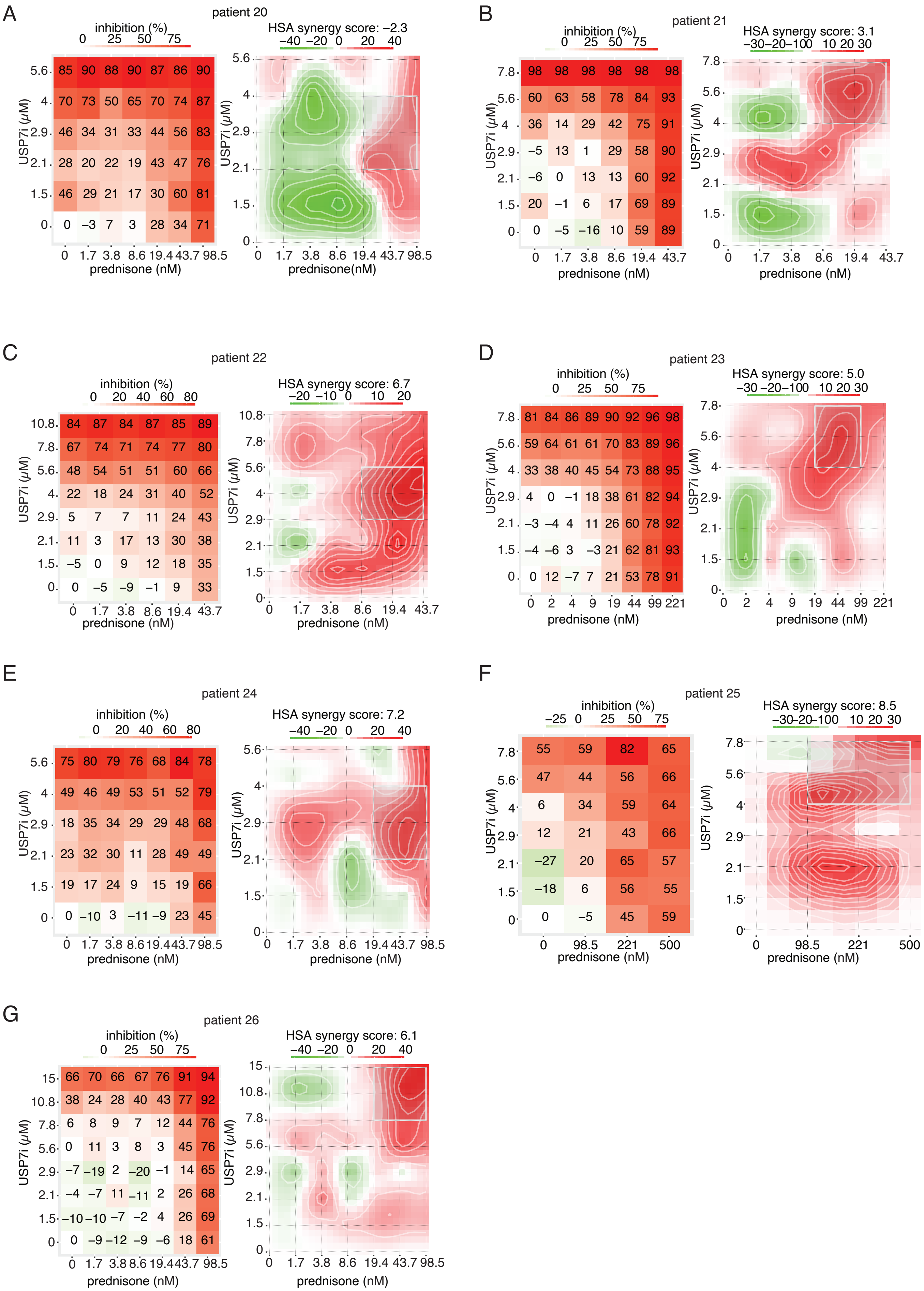

Supplementary Figure 12. Synergy between USP7i and prednisone in T-ALL patient samples. A-G, Synergy heatmaps for USP7 inhibitor and prednisone treatment for 5 days in 7 T-ALL patient samples. HSA analysis is shown.

Supplementary Figure 13. Jin, Gutierrez, et al., 2022

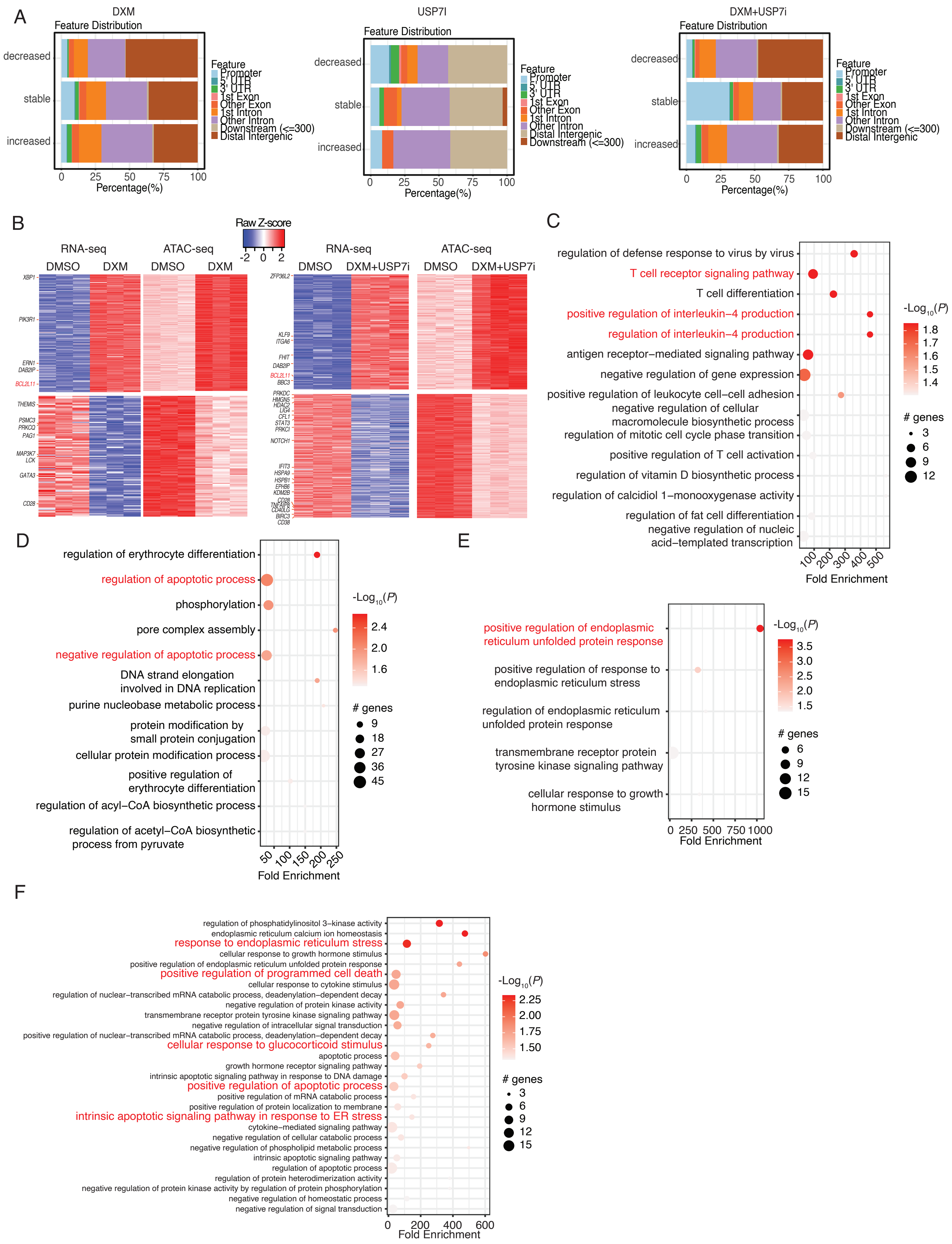

Supplemental Figure 13: Analysis of ATAC-seq and RNA-seq upon USP7i and glucocorticoid treatment. A, Genome distribution of ATAC-seq peak changes upon treatment with dexamethasone (DXM), USP7i, and their combination. **B,** Heatmap representation of RNA-seq and ATAC-seq changes in the dexamethasone group (left) and combination group (right) in DND41 cells. Labeled are transcripts presenting with overlapping changes in gene expression and chromatin accessibility. **C-D,** Gene Ontology analysis of down-regulated genes in the overlapping ATAC-seq and RNA-seq dataset upon dexamethasone treatment (**C**) or dexamethasone and USP7i treatments (**D**). **E-F,** Gene Ontology analysis of up-regulated genes in the overlapping ATAC-seq and RNA-seq datasets upon dexamethasone (**E**) or combination treatment (**F**).

Supplementary Figure 14. Jin, Gutierrez et al., 2022

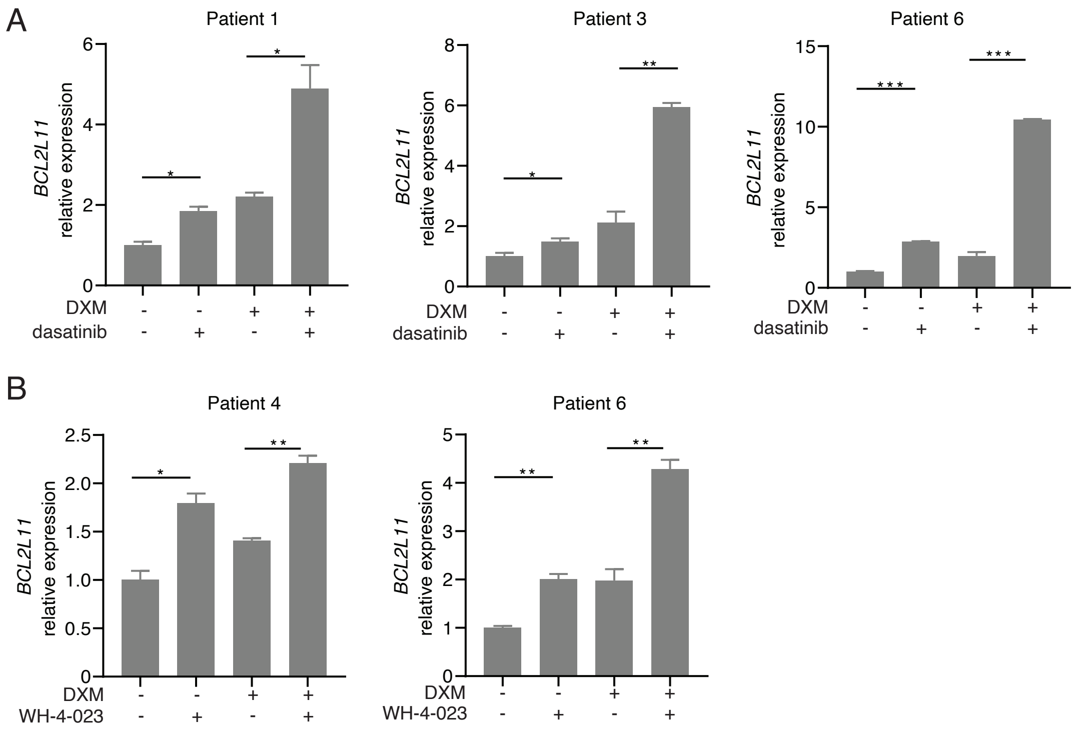

Supplementary Figure 14. LCK inhibition leads to up-regulation of *BCL2L11* upon glucocorticoid treatment in T-ALL patient samples. **A**, RT-qPCR analysis of *BCL2L11* in T-ALL patient samples treated with dexamethasone (DXM, 100 nM), dasatinib (2 μM), or their combination for 6 h (* $P<0.05$, ** $P<0.01$,). **B**, RT-qPCR analysis of *BCL2L11* in T-ALL patient samples treated with dexamethasone (100 nM), WH-4-023 (2 μM), or their combination for 6 h (* $P<0.05$, ** $P<0.01$, *** $P<0.001$).

Supplementary Figure 15. Jin, Gutierrez et al., 2022

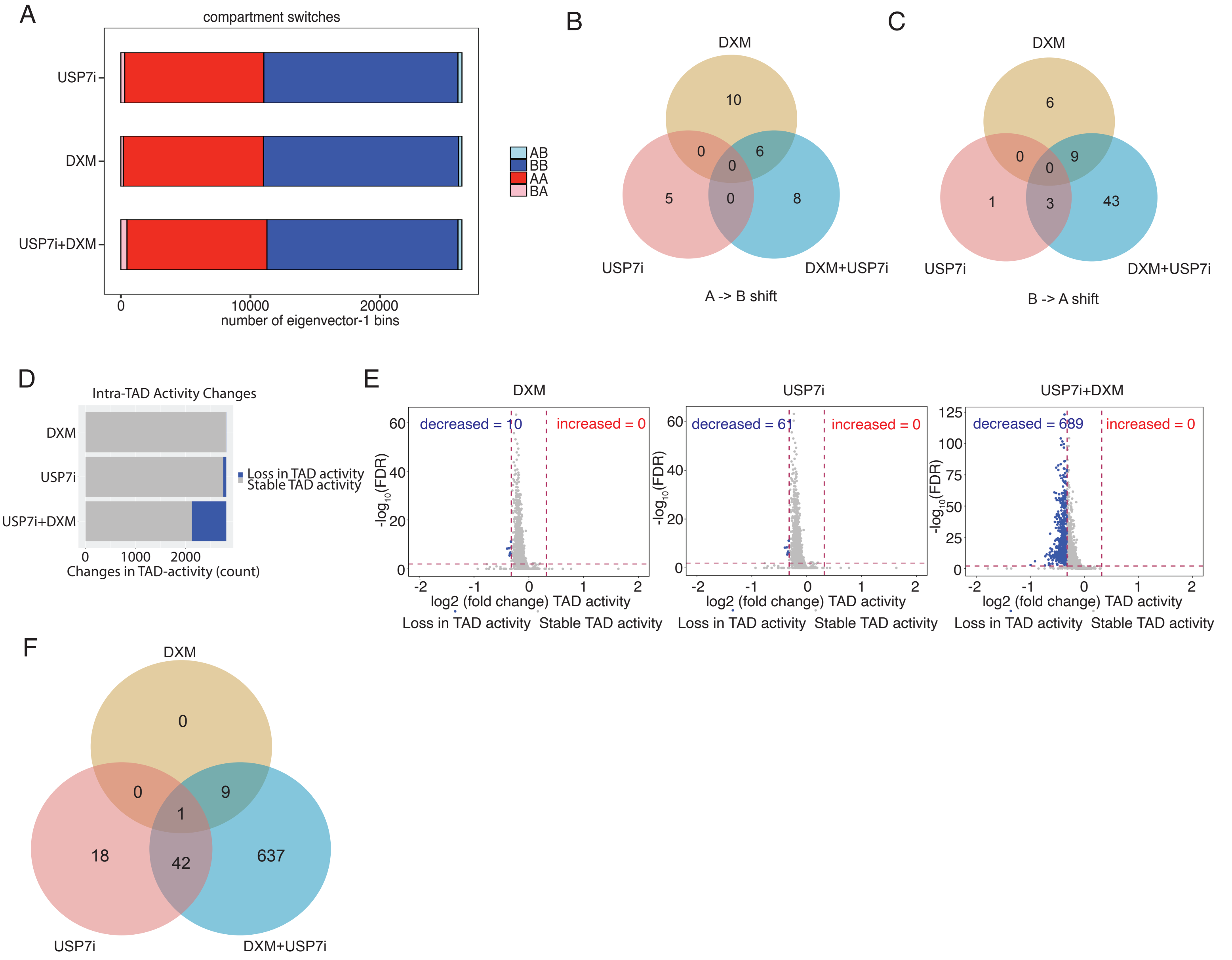

Supplementary Figure 15. 3D chromatin changes induced by glucocorticoids, USP7i, and their combination. **A**, Chromatin compartment status upon treatment with dexamethasone (DXM), USP7i, and their combination. **B-C**, Overlapping compartment changes (A→B or B→A) in the three treatment groups compared to vehicle treatment. **D-E**, Changes in intra-TAD activity in the three treatment groups compared to vehicle treatment. **F**, Overlap of changes in intra-TAD activity in the three treatment groups compared to vehicle treatment.

Supplementary Figure 16. Jin, Gutierrez et al., 2022

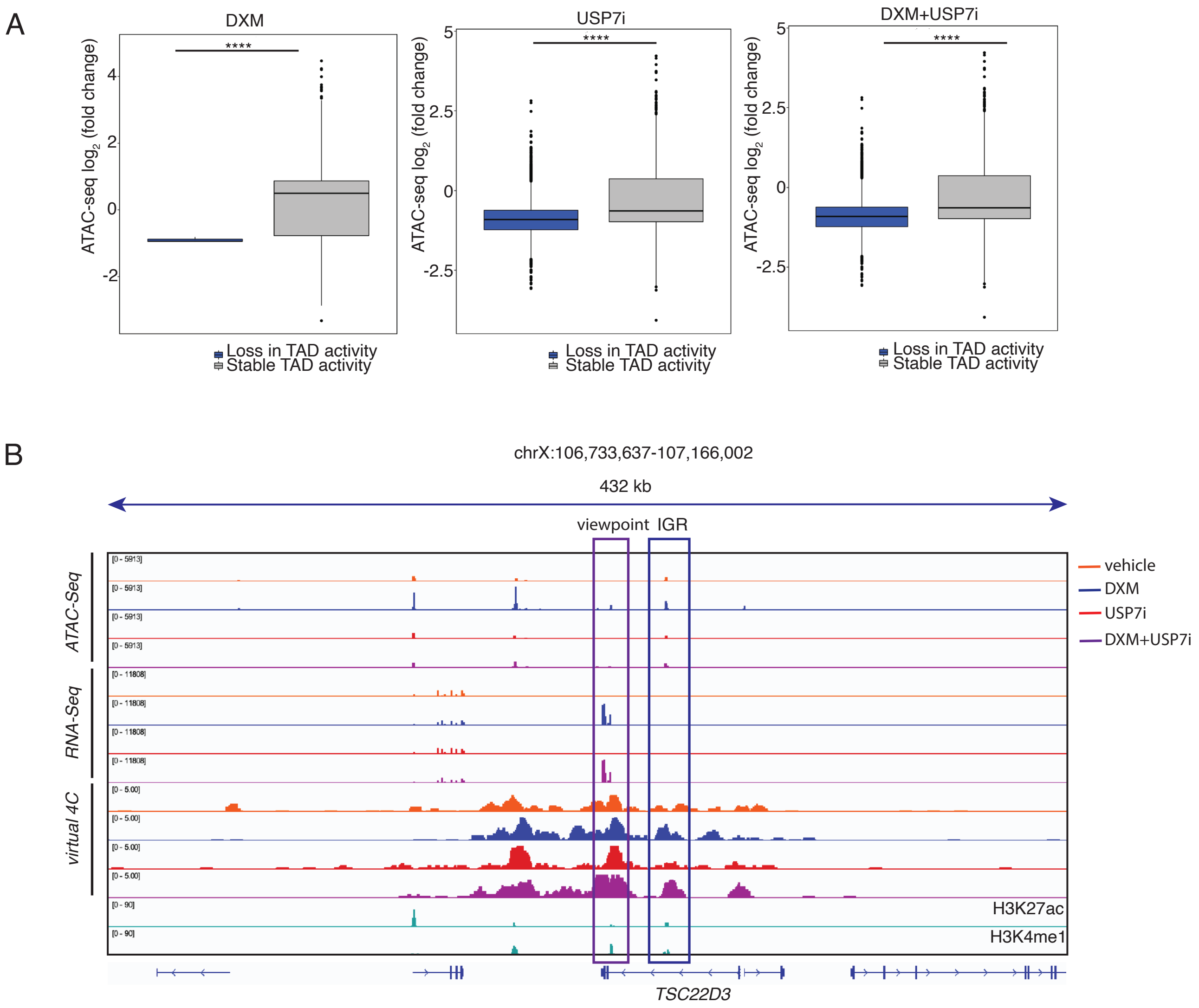

Supplementary Figure 16. Integrated analysis of gene expression, chromatin accessibility, and 3D chromatin changes induced upon glucocorticoid and USP7i treatment. A, Chromatin accessibility changes (ATAC-seq (compared to vehicle) in transcripts associated with lost or stable TAD activity upon dexamethasone (DXM), USP7i, or combination treatment. **B,** Snapshots of *TSC22D3* loci (*IGV browser*) presenting with gene expression (RNA-Seq), chromatin accessibility (ATAC-Seq), genomic interactions (virtual 4C), H3K27ac, and H3K4me1 status upon combination of glucocorticoids (DXM), USP7i or their combination. Viewpoint: promoter of *TSC22D3* (purple square); IGR: intronic GR-binding region (blue square).

List of Supplementary Tables

Supplementary table 1: USP11 IP-mass spectrometry analysis

Supplementary table 2: KεGG analysis upon *USP11* silencing and USP7 inhibition (USP7i)

Supplementary table 3: Proteomics and phosphoproteomics analysis upon *USP11* silencing

Supplementary table 4: Proteomics and phosphoproteomics analysis upon USP7i treatment (USP7i)

Supplementary table 5: Proteomics and phosphoproteomics analysis upon dasatinib treatment

Supplementary table 6: Patient information
